# Rapid Classification of Coffee Varieties Using Single-Bean Hot Gas Extraction Ion-Mobility Spectrometry with Machine Learning

**DOI:** 10.1021/acsmeasuresciau.6c00039

**Published:** 2026-04-02

**Authors:** Nathanael Aaron Prayoga, Chamarthi Maheswar Raju, Pawel L. Urban

**Affiliations:** Department of Chemistry, 150417 National Tsing Hua University, 101, Section 2, Kuang-Fu Rd., Hsinchu 300044, Taiwan

**Keywords:** adulteration, coffee bean, machine learning, rapid analysis, volatilome

## Abstract

Coffee is consumed by over one billion people daily, creating a high risk of adulteration where premium varieties are blended with inferior fillers. While various analytical techniques exist, they are often expensive and require labor-intensive sample preparation, such as grinding and brewing. Here, we present a proof-of-concept of a non-destructive platform that integrates ion-mobility spectrometry with online hot-gas extraction for rapid classification of coffee varieties using single-bean samples. The integration of a 1D convolutional neural network (CNN) model enables automated differentiation with 100% classification accuracy across four coffee bean varieties (Civet Arabica, Civet Robusta, carbonic maceration, and anaerobic fermentation natural) at an optimal bin width of 0.25 ms. When expanded to 10 diverse coffee bean varieties, the 1D CNN model achieved ∼92% independent test accuracy at a bin width of 0.10 ms. Furthermore, the system successfully monitored aroma degradation over 3 weeks and accurately predicted degradation patterns of anaerobic fermentation natural Arabica. Additionally, this 1D CNN model achieved 90% accuracy in predicting the presence of adulterants in coffee beans. Although the proposed method does not identify specific VOCs, the heuristic readouts it generates still enable rapid quality control and authenticity verification in commercial coffee beans.

## Graphical abstract

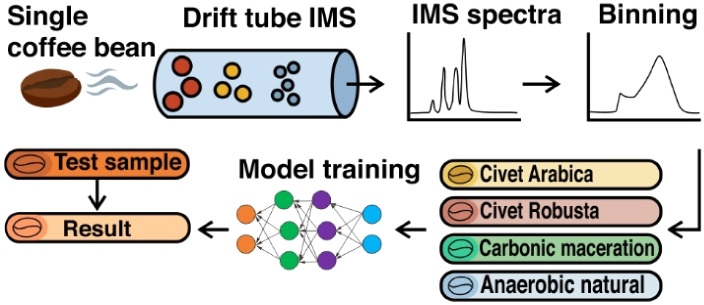

## 1 Introduction

More than 1 billion people consume coffee worldwide, appreciating its rich flavor and energizing effects.^1,2^ Arabica and Robusta are the two globally recognized coffee bean varieties, distinguished by their geographical distribution, physical characteristics, and flavor profile. However, Arabica coffee beans tend to be more expensive than other varieties for several reasons, such as flavor profile, specific growing conditions, use of labor-intensive harvesting methods, low yield, processing requirements, and greater market demand.^3^ On the other hand, Robusta coffee beans are more affordable than Arabica coffee beans due to their inferior flavor profile. They are frequently utilized in blends and instant coffee formulations.^4^ After harvesting, coffee producers utilize various processing methods to enhance the sensory profile of the coffee beans to align with the market demand. These methods include digestion, dry fermentation, wet fermentation, anaerobic fermentation, carbonic maceration, and the wine process.^3^

Coffee bean adulteration has emerged as a critical and escalating concern within the global market.^5–7^ This deceptive practice typically involves blending premium beans with inferior fillers, a strategy designed to obscure the visual defects of low-quality inventory and artificially inflate profit margins.^8,9^ Added to that, producers may dilute high-grade Arabica with cheaper, harsher Robusta beans, or hide insect-damaged, black, or moldy beans within a bulk shipment, relying on the consumer’s inability to distinguish them at a glance.^9–11^ In more extreme cases, producers add coffee husks and sticks, corn, barley, brown sugar, and soy to adulterate the roasted coffee beans, ultimately resulting in a significantly compromised flavor profile and potential health risks.^7^

Over the years, various analytical methods have been developed to classify different types of green and roasted coffee beans and detect their adulteration. These methods employ a range of analytical instruments, including ultraviolet–visible (UV–vis) spectroscopy,^12^ Fourier transform infrared (FTIR) spectroscopy,^13^ near-infrared (NIR) hyperspectral imaging,^14^ headspace (HS) gas chromatography (GC) ion-mobility spectrometry (IMS),^15^ paper spray mass spectrometry (MS),^16^ and single-bean MS with a machine learning strategy.^17^ For example, dos Santos et al. developed a UV–vis spectroscopy method to authenticate the different origins of green coffee beans from Cerrado Mineiro, Brazil.^12^ In this approach, liquid extracts of green coffee varieties were analyzed within the wavelength range of 230–450 nm using UV–vis spectroscopy, and the resulting spectra were classified employing chemometric multivariate techniques.^12^ In other work, Lyman et al. developed an FTIR spectroscopy method to classify brewed roasted coffee into light, medium, and dark roast categories based on changes in the concentration of carbonyl compounds.^13^ Later on, Cestari developed a simple method to classify pure and adulterated Arabica coffee utilizing attenuated total reflectance (ATR) FTIR spectroscopy.^9^ In this approach, 0.5 g of finely ground coffee powder was subjected to 32 scans to obtain ATR-FTIR spectra. Subsequently, the resulting spectra were processed and analyzed using discriminant analysis software. Furthermore, Forchetti and Poppi developed a NIR hyperspectral imaging-based method for detecting and quantifying adulterants in roasted and ground coffee.^14^ This approach utilizes NIR hyperspectral imaging in conjunction with the multivariate curve resolution alternating least-squares algorithm to detect and identify adulterants such as coffee husks, wood fragments, and soil in coffee samples.^14^

Konieczka et al. introduced methodologies employing direct HS-GC-IMS and activated charcoal strip (ACS) HS-GC-IMS characterization of Arabica and Robusta coffee.^15^ Their approach involves preconcentration of HS volatile organic compounds (VOCs) from coffee using ACS, followed by HS-GC-IMS analysis. The proposed methodology enables the characterization of the Arabic and Robusta coffee varieties based on their VOCs profile. Conversely, Pumbua et al. utilized paper spray MS as a tool to classify coffee based on their geographical origins.^16^ In this technique, triangular papers with millimeter-scale dimensions are immersed in the grounded coffee extracts, removed, and air-dried to prepare them for paper spray MS analysis. After that, the resulting MS data were then classified using principal component analysis and linear discriminant analysis.^16^ Recently, Tsai et al. developed a single-bean MS technique incorporating a machine-learning approach to distinguish Kopi Luwak coffee from other varieties.^17^ This method involves depositing a microsized solvent droplet onto the coffee bean surface to initiate a Taylor cone, induced by the potential difference between the MS inlet and the coffee bean. The acquired MS spectra were analyzed and classified using a neural network-based machine learning approach.^17^

Non-destructive sample analysis is an environmentally friendly method that preserves sample integrity, minimizes the risk of contamination, and optimizes cost-effectiveness.^18^ Previously reported UV–vis and FTIR spectroscopy methods required labor-intensive sample preparation procedures, including grinding, performing extraction, and brewing processes.^12,13^ Moreover, coffee is a complex matrix composed of hundreds of compounds, which can produce intricate UV–vis and FTIR spectra, posing significant challenges for analytical evaluation. Although NIR hyperspectral imaging is a powerful tool for detecting adulterants in coffee, it presents challenges related to sample preparation and environmental factors.^19^ Parameters—such as moisture, particle size, roasting levels, condition, temperature, humidity, and lighting—must be controlled carefully to ensure optimal performance.^19^ Conversely, HS-GC-IMS offers high sensitivity and selectivity, as well as the advantages of non-destructive analysis of coffee products. However, the typical analysis time of HS-GC-IMS is 15–30 min per sample, which can reduce the overall sample throughput.^15,20^ On the other hand, MS is a powerful and versatile—but very expensive—analytical technique for detecting and quantifying diverse chemical compounds. To operate, MS requires an ionization method to convert chemical compounds into ions before detection. In the context of applying the coffee extraction to a paper spray ionization strip, the process involves grinding, brewing, and extracting coffee beans to enable the identification of associated chemical compounds.^16^ These steps are labor-intensive and decrease the overall throughput of the sample analysis.

Here, we aim to develop an alternative simple, rapid, and inexpensive analytical approach for the identification of coffee varieties. The approach takes advantage of hot gas extraction coupled online with ion-mobility spectrometry (Figure 1). The analysis is followed by data treatment using machine learning. The sample size is as small as a single coffee bean, which enables differentiation of mixed coffee beans of different varieties.

**Figure 1:**
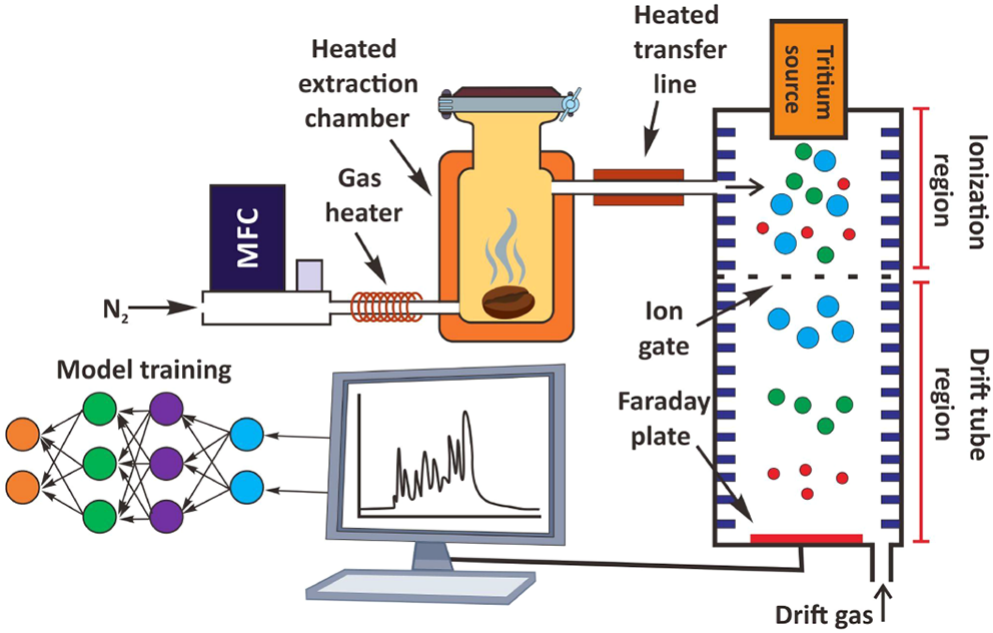
Schematic diagram for the hot gas extraction ion-mobility spectrometry of a single coffee bean.

## 2 Materials and Methods

### 2.1 Samples and Sample Preparation

Civet Arabica (process: digestion; roast: medium; origin: Indonesia), Civet Robusta (process: digestion; roast: medium-dark; origin: Indonesia), carbonic maceration Arabica (process: carbonic maceration; roast: medium; origin: Indonesia), and fully washed Arabica (process: washed; roast: medium; origin: Indonesia) coffee beans were all sourced from Aceh (Sumatra Island, Indonesia) to minimize regional variability. Anaerobic fermentation natural Arabica (process: anaerobic fermentation natural; roast: medium; origin: Indonesia) coffee beans were sourced from Jambi, Sumatra Island, Indonesia. Columbia Arabica (CDD) (process: decaffeinated; roast: dark; origin: Columbia), Costa Rica Arabica (CBL) (process: black honey; roast: light; origin: Costa Rica), Ethiopia Arabica (ENM) (process: natural; roast: medium; origin: Ethiopia), fruit blend Arabica (FXM) (process: unknown; roast: medium; origin: Ethiopia and Costa Rica), Guatemala Arabica (GWMD) (process: washed; roast: medium-dark; origin: Guatemala), Italy commercial blend Arabica (IXD) (process: unknown; roast: dark; origin: unknown), Indonesia Robusta (IRWD) (process: wet hull; roast: dark; origin: Indonesia), Uganda Arabica (UAL) (process: anaerobic fermentation natural; roast: light; origin: Uganda), Taiwan Alishan Arabica (TWM) (process: washed; roast: medium; origin: Taiwan), and Yemen Arabica (YNM) (process: natural; roast: medium; origin: Yemen), coffee beans were purchased from local shops in Hsinchu, Taiwan. To prevent the degradation of VOCs in the coffee beans during the analysis period, the roasted coffee beans were divided into 15 small portions. Each portion was stored in nitrogen-filled, airtight aluminum foil pouches (15 × 8 cm), and kept at 4 °C in a refrigerator.

To optimize the parameters of the single-bean hot gas extraction IMS setup, fully washed Arabica coffee beans were ground using a Timemore Chestnut C2 coffee grinder (Timemore, Shanghai, China) with a 15-click adjustment. This ground coffee powder was divided into portions weighing 170 ± 10 mg, which were packed in tea filter bags for optimization purposes.

### 2.2 Setup for Hot Gas Extraction Sampling of a Single Coffee Bean

In this setup, a mass flow controller (model no: F-201CL-013–1K0-A; max flow rate: 1 L min^–1^; Bronkhorst, Ruurlo, The Netherlands) was utilized to measure and control the flow rate of nitrogen for extraction (Figure S1A). The mass flow controller was connected to an electric gas heater (electric heating tube; 150 V; 500 W; Ching Ta Heating Company, Taoyuan, Taiwan; Figure S1B) using stainless steel 1/4-in. Swagelok tube fittings and a 56.5 cm transparent PTFE tube (OD: 6 mm; ID: 4 mm; Figure S1H). Furthermore, the electric gas heater was connected to an in-house-built airtight temperature-controlled sample chamber (fabricated by the NTHU workshop, Hsinchu, Taiwan) for extracting VOCs from individual coffee beans (Figures S1C,E and S2). A 1/8-in. male connector was used for this connection. A stainless steel tube (OD: 3 mm; ID: 1.5 mm) was incorporated in order to prevent condensation of the extracted VOCs during their transfer to the IMS module (see below; Figure S1D,F,G,I). Furthermore, this stainless steel transfer tube was heated using 4 custom-built temperature-controlled heating plates fabricated by the NTHU workshop (Hsinchu, Taiwan; Figures S1D,F,G,I and S3). One end of the transfer tube was connected to the VOCs extraction chamber using a stainless steel Swagelok tube fitting 1/4-in. to 1/4-in. union (Figure S1F,G,I). The other end was linked to the IMS with another stainless steel Swagelok fitting, featuring a reduced union for a smooth transition from 1/8-in. to 1/16-in. (Figure S1F,G,I). Additionally, the flow rate of the mass flow controller was regulated using an in-house-built circuit (Figure S4). In this circuit, there are two button modules. One button controlled the nitrogen flow rate at 0.2 L min^–1^ for a sampling time of 1 min. The other button was used to pump nitrogen at a flow rate of 0.5 L min^–1^ for a cleaning duration of 5 min. During the sample analysis, the IMS module and mass flow controller were operated manually.

### 2.3 Ion-Mobility Spectrometry

The IMS module (OEM-IMS; G.A.S., Dortmund, Germany) was equipped with a tritium-based radioactive atmospheric pressure chemical ionization source, with an initial activity of approximately 300 MBq. It featured a 98 mm drift tube and a Faraday plate detector. Prior to introduction, nitrogen drift gas was passed through a moisture trap (catalog no. 20618; Supelco, Sigma-Aldrich, St. Louis, MO, USA).

The default instrumental settings were: positive drift voltage polarity, injection pulse width of 150 μs, spectrum acquisition time of 20 ms, drift voltage of 240 V, blocking voltage of 120 V, injection voltage of 2500 V, aperture voltage of 0 V, drift gas flow rate of 150 mL min^–1^, and drift tube temperature of 80 °C (idle at 40 °C).

### 2.4 Data Processing

The IMS module is operated with standalone software (version 01.02; G.A.S.) to acquire and store raw IMS measurement data (note: data values are in mV) in MEA files, a proprietary binary format. Please refer to the flowchart (Figure S5) for an overview of the data processing. The data files were processed using VOCal software (version 0.1.1; G.A.S.) to export CSV files used in further analysis. A CSV file contains 2,999 columns, each data point representing 0.006667 ms, resulting in a drift time or spectrum acquisition time of 20 ms (0.006667 ms per data point × 2,999 data points ≈ 20 ms drift time). It also includes 143 rows, corresponding to a total runtime of 60 s. These CSV files were imported into a custom-designed graphical user interface (GUI) to compute the average for each column (Figure S6). This GUI also allows for the visualization of ion-mobility spectra (mobiligrams) as a graph plotting drift time against the average intensity in mV, for the chosen drift time range in ms (Figure S6). This average intensity was saved by pressing the button “Save CSV”. The saved CSV file was subsequently imported into the “Drift Tube IMS Data Binning Viewer” GUI for the purpose of binning the data (Figure S7). Binning converts continuous drift-time signal intensities into fixed-width intervals, reducing data complexity and enabling reliable statistical and machine-learning analysis.^21,22^ Proper binning enhances comparability between samples by smoothing out small instrumental fluctuations while maintaining chemically significant features.^23^ However, selecting the correct bin width is crucial, as too fine binning can increase noise, while too coarse binning can cause peaks to merge and important discriminative information to be lost.^24,25^ To optimize the binning process, we divided the drift time range from 8.0 to 15.0 ms into bins of varying widths and excluded the reactant ion peak (RIP) during binning. The RIP (H^+^(H_2_O)_*n*_ in positive mode)^26^ was excluded because it is not analyte-specific and mainly reflects instrumental and environmental conditions, which could mislead data analysis. Including it skews intensity scaling, causing models to learn RIP fluctuations instead of true analyte mobility features. Ion-mobility spectra were binned by grouping data points with floor division of the acquisition index, using fixed bin sizes. The selected bin sizes were 15.01 (0.1 ms), 22.52 (0.15 ms), 30.03 (0.20 ms), 37.54 (0.25 ms), and 45.04 (0.3 ms). For each bin, mean drift time and signal intensity were calculated, thereby reducing the data’s dimensionality. The use of a noninteger bin size allowed for precise control of the final feature vector length while maintaining chemically relevant spectral information across all samples. This resulted in 70 bins for 0.1 ms, 47 for 0.15 ms, 35 for 0.20 ms, 28 for 0.25 ms, and 23 for 0.30 ms. These bins were organized in Excel (version LSTC Professional 2021; Microsoft, Redmond, WA, USA) and saved as a CSV file to create a data set or sample data. In order to evaluate the variability of the binned ion mobility (fingerprint) spectra for Civet Arabica (Figure 2A), Civet Robusta (Figure 2B), carbonic maceration Arabica (Figure 2C), and Anaerobic fermentation natural Arabica (Figure 2D), we selected one bin point (the ninth bin in the data with a 0.25 ms bin width) and calculated the RSD, which are 7.95% (*n* = 36), 6.00% (*n* = 36), 4.14% (*n* = 36), and 4.99% (*n* = 36), respectively.

**Figure 2:**
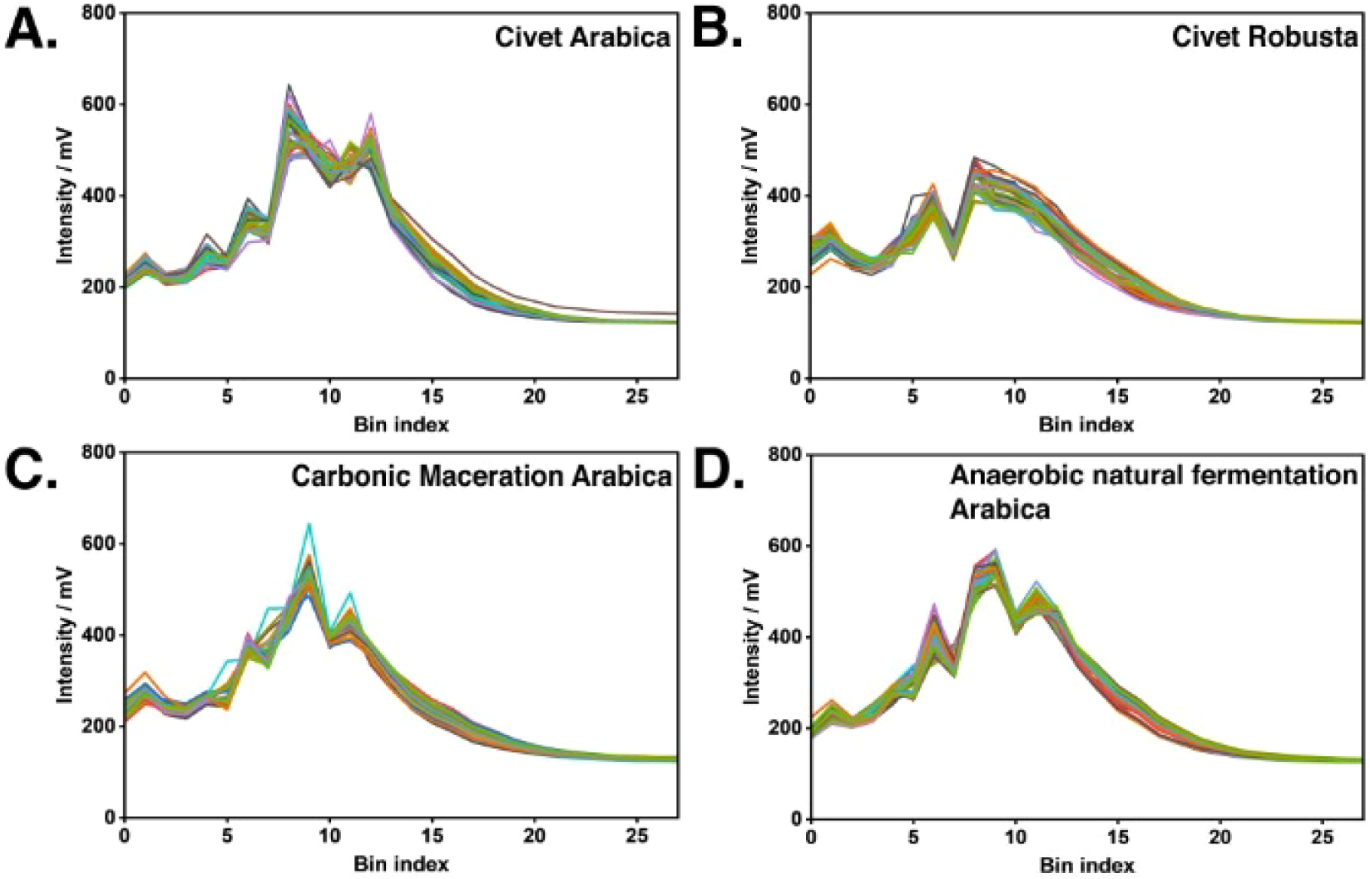
Overlaid 36 binned ion-mobility spectra (bin width: 0.25 ms) for training the CNN model: (A) Civet Arabica; (B) Civet Robusta; (C) carbonic maceration Arabica; (D) anaerobic natural fermentation Arabica.

### 2.5 Machine Learning

The GUI was developed using Python (version 3.10.18; The Python Software Foundation, DE, USA) and the Tkinter library.^27,28^ The GUI allows users to interactively upload data sets, train a convolutional neural network (CNN), and test its predictions (Figure S8). The workflow starts with the user selecting a training data set, and optionally, a test data set via file dialogue windows. The training data set is read from a CSV file, where the last column is treated as the label (representing single coffee bean classes), and the remaining columns are considered numerical features. The labels are encoded into a categorical format using the “LabelEncoder” function and “to_categorical”.^29–31^ Meanwhile, the features are normalized using the “StandardScaler” function.^32^ To make it compatible with a 1D CNN, the features were reshaped into a three-dimensional format (samples, features, 1).^33,34^ The data set was split into training and validation sets at a 80:20 ratio randomly.^35^ A CNN was designed with a series of stacked convolutional and pooling layers, along with dense hidden layers (please refer to Table S1 for model summary). Dropout was used for regularization, and a “softmax” function was applied in the output layer to predict multiple classes.^36^ The model was trained for 300 epochs, iterating through the entire training data set 300 times to adjust the weights and minimize the loss function.^37^ During this process, intermediate updates were not displayed, but the training and validation losses and accuracies were recorded in a textbox for later visualization.

The prediction function enabled users to upload either a separate binned test data set or individual binned sample data. This data was then normalized using the same “StandardScaler” function and reshaped into a format suitable for a 1D CNN before being input into the trained CNN model. The model generated a probability distribution for each class for each sample, and the predicted class was determined by selecting the class with the highest probability.

## 3 Results and Discussion

### 3.1 Optimization of the Single-Bean Hot Gas Extraction IMS

In order to optimize the operating and extraction conditions of the single-bean hot gas extraction IMS setup, the drift gas flow rate, drift tube temperature, injection pulse width, extraction (nitrogen) flow rate, extraction chamber and gas heater temperature, and transfer line temperature were varied, one parameter at a time. When the drift gas flow rate was varied from 210 to 290 mL min^–1^, we observed a similar signal response and fingerprint up to 250 mL min^–1^ (Figure S9). Nevertheless, when the flow rate was increased to 270 mL min^–1^, the signal intensity declined. Thus, we selected a flow rate of 250 mL min^–1^. When the drift tube temperature rose from 40 to 80 °C, we observed that the drift times of the reactant ion peak decreased (Figure S10). This can be attributed to a reduction in gas density and the increase in ion velocity.^38^ However, there were no significant changes in the signal intensity or fingerprint pattern as the temperature increased. To avoid carryover effects, we selected 80 °C as the drift tube temperature.

When the injection pulse width was varied from 110 to 190 μs, we observed a decrease in the signal intensity and a change in the fingerprint pattern at 110 μs (Figure S11). Nonetheless, from 130 to 190 μs, there were no significant changes in either the fingerprint pattern or the signal intensity (Figure S11). An injection pulse width of 150 μs can be considered the optimal value, given satisfactory peak separation and signal intensity. The extraction gas (nitrogen) flow rate was varied from 100 to 500 mL min^–1^. A signal corresponding to the moisture peak was only detected at the 100 mL min^–1^ extraction gas flow rate (Figure S12). At flow rates between 200 and 500 mL min^–1^, we observed a fingerprint pattern of grounded coffee. A flow rate of 200 mL min^–1^ was chosen to prevent peak broadening and minimize carryover.

Although it is possible to set the extraction chamber and gas heater temperatures independently, we maintained similar temperatures for both to minimize fluctuations in the extraction chamber’s temperature. When the temperatures of the extraction chamber and gas heater were varied simultaneously from the room temperature (∼24 °C) to 60 °C, we observed several low-intensity peaks at room temperature that were not well separated (Figure S13). As the temperature rose from 30 °C up to 60 °C, the signal intensity of the fingerprint features increased. Nonetheless, we selected 50 °C as the temperature for both the extraction and the gas heater to avoid degradation of the fingerprint pattern peaks. As the transfer line temperature was varied from 50 to 90 °C, we observed a decrease in the intensity of the fingerprint pattern peaks at 50 °C. At 80 and 90 °C, the intensities of the fingerprint pattern peaks reached their maxima (Figure S14). However, some of the peaks decreased at the higher temperatures. Therefore, 70 °C can be regarded as the optimal temperature for achieving a reasonable intensity and profile of the fingerprint pattern peaks. The optimized operating parameters for single-bean hot-gas extraction IMS are listed in Table S2.

To determine the necessary sample size of coffee beans for our analysis, the sample size was varied from 1 to 4 beans. For this optimization, anaerobic fermentation natural Arabica coffee beans were used. In the ion-mobility spectra of 2 coffee beans, peak 1 showed an increase compared to the ion-mobility spectra of 1 coffee bean, while peak 2 nearly disappeared (Figure S15). However, moving from the ion-mobility spectra of 2 coffee beans to those of 4 coffee beans, we did not observe any clear changes, indicating signal saturation. To prevent saturation and minimize carryover, we have opted for analysis of single coffee beans in the subsequent experiments.

### 3.2 Training the Initial Machine Learning Model to Distinguish Four Coffee Varieties

To train and validate the 1D CNN model, we collected 144 single-bean ion-mobility spectra from four varieties of coffee beans: Civet Arabica, Civet Robusta, carbonic maceration Arabica, and anaerobic fermentation natural Arabica (Figure 2). These varieties were selected due to the common origin and distinct ion-mobility spectral patterns they yielded. The data was gathered over 3 days, with 12 ion-mobility spectra recorded each day to enhance data robustness. The ion-mobility spectral data for four coffee bean varieties were processed and binned using bin widths of 0.10, 0.15, 0.20, 0.25, and 0.30 ms, as shown in Figure S5. The binned data were organized in an Excel file and imported into a custom-built 1D CNN GUI (Figure S8). Eighty percent of the binned ion-mobility spectra was used for training. The remaining 20 percent was randomly selected using the “random Python library”^39^ and reserved for validating the model’s accuracy. The obtained accuracies were 93.10%, 93.10%, 96.55%, 100.00%, and 96.55%, for bin widths 0.10, 0.15, 0.20, 0.25, and 0.30 ms, respectively (Figures S16 and S17). Interestingly, with 0.25 ms bin width, all the samples were successfully classified (Figures S16 and S17). Accuracy varies with bin width because temporal binning alters the signal-to-noise ratio and the effective temporal resolution of the IMS features that the 1D CNN learns. Smaller bins preserve fine peak structure but retain noise, while moderate bins optimally smooth noise without destroying discriminative peak relationships, whereas larger bins begin to oversmooth class-specific features, reducing generalization. Furthermore, the training loss after 300 epochs (0.00) was exceptionally low for a 0.25 ms bin width 1D CNN model, indicating that the model fits the training data almost perfectly (Figure S8). Validation accuracy of 100% was achieved, indicating that the predictions for the validation samples were correct (Figures S16 and S17). Additionally, the validation loss is very close to zero (0.0005), indicating that the model is not only accurate but also highly confident in its predictions (Figures S8 and S18). The high accuracy in training and validation was achieved due to distinct drift-time patterns and nonoverlapping features across the four types of single coffee bean data.

To assess the 1D CNN model’s performance across various coffee bean batches, we bought a new batch of anaerobic fermentation natural Arabica coffee beans from the same supplier, three months after our initial purchase. After that, we generated three fingerprints (IMS spectra) of anaerobic fermentation natural Arabica using single-bean hot gas extraction IMS and considered these as three replicates for the test data set. This test data set is processed and binned at 0.25 ms, as shown in the flowchart (Figure S5). After that, this processed and binned data set was imported into the 1D CNN model GUI (Figure S8) for predictions. We observed that the trained 1D CNN model successfully predicted the three replicates as anaerobic fermentation of natural Arabica with 100% probability (*vide infra*).

### 3.3 Degradation Study

It is known that the aroma of coffee beans deteriorates over time due to different processes such as oxidation,^40^ volatilization and evaporation,^40,41^ hydrolysis and polymerization,^42^ light-induced degradation,^43^ and temperature effects.^40^ Developing a rapid analytical method to assess the freshness of coffee beans would be advantageous. The approach outlined above provides a suitable solution. To conduct the degradation test, three airtight ziplock aluminum foil pouches were used, each containing 40 anaerobic fermentation natural Arabica coffee beans. On the first day of the test, the beans from all the three pouches were analyzed to evaluate their freshness and obtain a fingerprint of the coffee beans (Figure 3A,B,C). Subsequently, the first pouch was flushed with nitrogen, the second pouch was sealed airtight, and a ∼1 cm-wide notch was made in the third pouch to expose the coffee beans to the atmospheric air (Figure S19). All three pouches were then stored at room temperature to replicate typical household conditions. To assess the degradation of VOCs, coffee beans were tested weekly for a duration of 3 weeks using single-bean hot gas extraction IMS fingerprinting. Upon analyzing the fresh coffee beans (week 0) from three pouches, we observed that the fingerprints were similar to those previously analyzed, and these fingerprints were successfully classified as anaerobic fermentation natural Arabica using a 1D CNN model (Figure 3A–C and Table S3). After 1 week, the test was repeated to assess the degradation of VOC levels in the nitrogen-flushed pouch. A slight decrease in intensity was observed at peaks 1, 2, and 3 (Figure 3A). The obtained data were successfully classified using a 1D CNN model, with probabilities ranging from 99.96% to 100.00%. During the second and third weeks, noticeable changes appeared in the fingerprint pattern. By then, the 1D CNN model could not classify these changes, leading to false predictions that missed detecting anaerobic fermentation in natural Arabica. This suggests a substantial loss of VOCs in the coffee beans, with probabilities reported at 0.00% (Figure 3A and Table S3). When analyzing coffee stored in the airtight-sealed pouch for 1 week, we noticed that peak 1 disappeared, while the intensity of peak 2 decreased. The remaining peaks also showed a slight decrease. The sample was classified as an anaerobic fermentation natural process, with probabilities ranging from 94.29% to 100.00% (Figure 3B and Table S3). By the second week, all peak intensities continued to decline, and by the third week, the total fingerprint pattern had changed significantly, making it impossible for the 1D CNN model to recognize it, resulting in false predictions (Figure 3B and Table S3). During analysis of coffee beans stored in the improperly sealed pouch, we observed that peaks 1 and 2 disappeared in the first, second, and third weeks, leading to a significant change in the spectral patterns (Figure 3C). Consequently, the 1D CNN model failed to classify the data correctly, produced false predictions, and was unable to detect anaerobic fermentation in natural Arabica, with reported probabilities of 0.00% (Figure 3C and Table S3). These results indicate that nitrogen-filled, airtight coffee bean pouches maintain freshness longer than improperly sealed options. Furthermore, the above findings demonstrate that the single-bean hot gas extraction IMS method is effective for assessing coffee aroma freshness.

**Figure 3:**
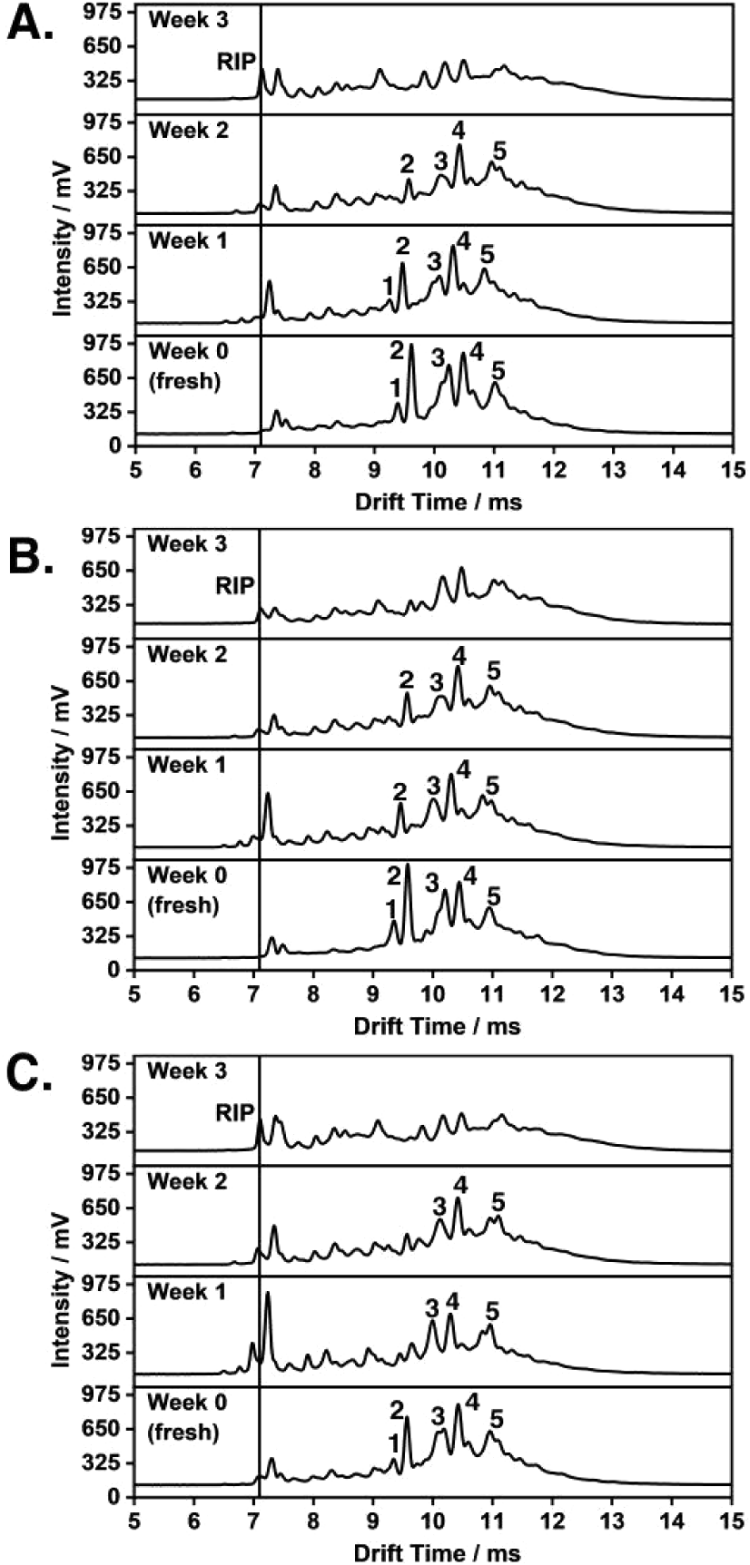
Degradation test of VOCs in coffee beans: (A) nitrogen-flushed and sealed coffee beans; (B) airtight sealed coffee beans; (C) improperly sealed coffee beans. RIP – reactant ion peak.

### 3.4 Adulteration Study

The VOCs such as alcohols, aldehydes, ketones, phenols, pyrazines, pyridines, pyrroles, and sulfur compounds, determine the sensory properties of coffee.^44^ Moreover, thiols significantly affect coffee quality and flavor, even in low concentrations, due to their extremely low odor threshold.^45,46^ To evaluate the applicability of the developed method in adulteration testing, 75% Civet Arabica coffee beans (15 beans) were mixed with 25% Civet Robusta (5 beans) in airtight ziplock aluminum foil pouches (15 cm × 8 cm), and incubated at room temperature for 1 h to adulterate the Civet Arabica coffee beans with Civet Robusta. After 1 h, we acquired the ion-mobility spectral fingerprints of these 20 coffee beans (15 Civet Arabica and 5 Civet Robusta). Interestingly, we observed that Civet Arabica and Civet Robusta exhibited similar ion-mobility spectral fingerprints (Figure 4A and Table S4). When testing these ion-mobility fingerprints with a 1D CNN model, it predicted that the probability of Civet Arabica was zero in 18 fingerprints and 100% in 2 fingerprints, indicating false positives (Figure 4A and Table S4). Overall, the 1D CNN model achieved 90% accuracy in the adulteration test. This observation suggests that the total gas-phase VOCs from Civet Arabica and Civet Robusta do not merely combine; instead, they undergo rebalancing through diffusion and adsorption processes. Such behavior is consistent with previous findings demonstrating selective adsorption and competitive interactions of VOCs on coffee matrices.^47^ These processes occur between the porous surfaces of the beans and their oils, resulting in a noticeable change in aroma.^44,48,49^

**Figure 4:**
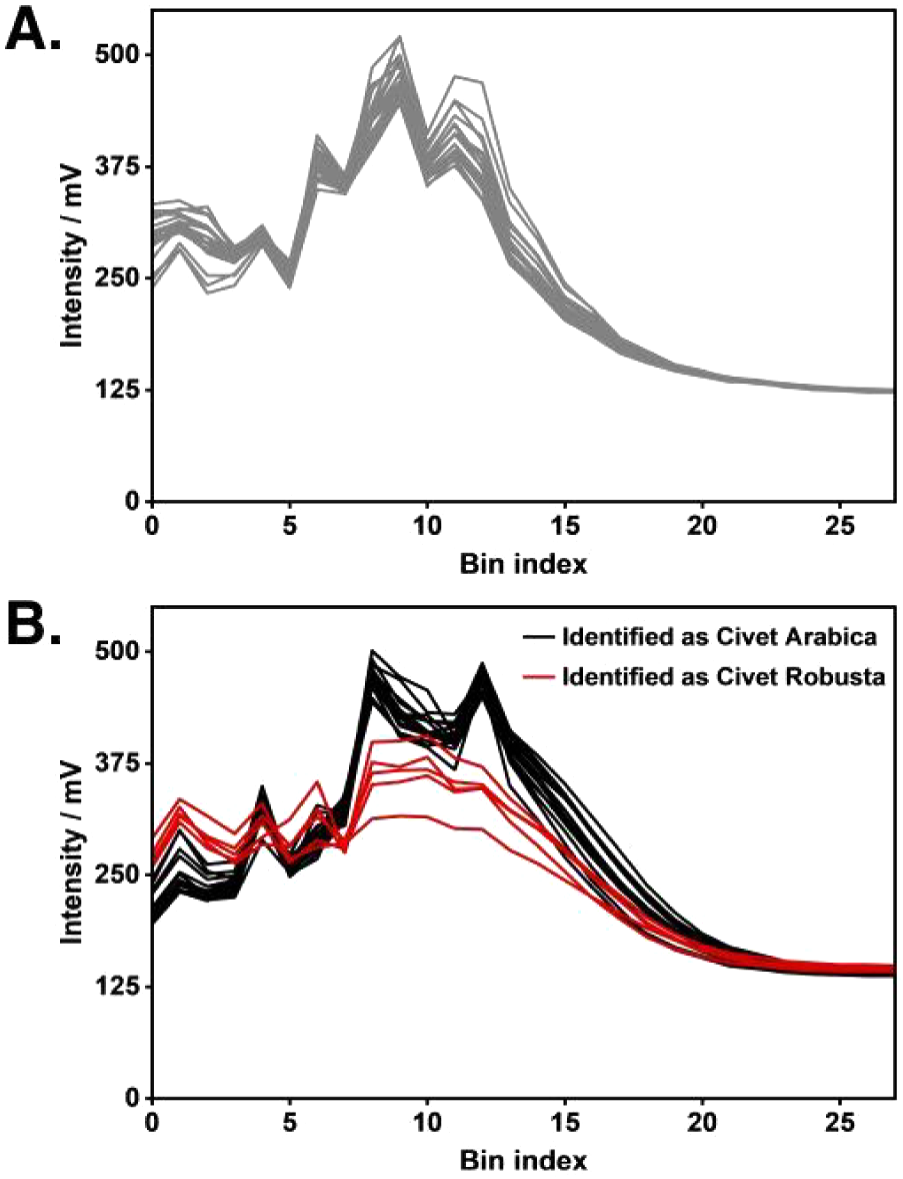
Adulteration test binned ion-mobility spectra: (A) uncovered coffee beans (75% Civet Arabica {15 beans} and 25% Civet Robusta {5 beans}); (B) aluminum foil-wrapped coffee beans (75% Civet Arabica {15 beans} and 25% Civet Robusta {5 beans}).

To confirm the diffusion and adsorption process of headspace VOCs in coffee beans, 15 Civet Arabica beans (75%) were individually wrapped in two 3 × 3 cm pieces of aluminum foil, and 5 Civet Robusta beans (25%) were wrapped in the same manner. The packed beans were then combined in airtight ziplock aluminum foil pouches (15 cm × 8 cm), and incubated at room temperature for 1 h. This time, we were able to record single-bean ion-mobility spectra of two kinds corresponding to the two coffee types (Figure 4B). Additionally, our 1D CNN model successfully classified all Civet Arabica and Civet Robusta coffee beans (Figures S1J), with probabilities ranging from 89.84% to 100.00% for Civet Arabica, while Civet Robusta had zero probability (Figure 4B and Table S4). Overall, the 1D CNN model achieved 100.00% accuracy in this test. These results show that single-bean hot gas extraction IMS, combined with a 1D-CNN model, can effectively detect adulteration in coffee beans.

### 3.5 Classification of 10 Coffee Bean Varieties with Different Origins, Processing Methods, and Roasting Levels Using Single-Bean Hot Gas Extraction IMS and 1D CNN Model

To further demonstrate the capabilities of a single-bean hot-gas-extraction IMS employing a 1D CNN model, we selected 10 coffee bean varieties from diverse origins, processing methods, and roasting levels, specifically CDD, CBL, ENM, FXM, GWMD, IXD, IRWD, UAL, TWM, and YNM (please refer to section 2.1 for the full descriptions of the coffee varieties). To train and validate the 1D CNN model, we collected 10 ion-mobility spectra (fingerprints) for each coffee variety and binned them using five bin widths—0.1 ms (Figures 5 and S20), 0.15, 0.20, 0.25, and 0.30 ms—to assess the effect of binning on 1D CNN model accuracy. Interestingly, we attained 100% classification accuracy across all bin widths when utilizing 80% of the data for training and 20% for validation, with random selection conducted via the “random Python library”. Furthermore, to test the trained 1D CNN model, an independent test data set was created. This data set included four selected coffee types (CBL, CDD, IRWD, and IXD). These varieties were analyzed on a different day, with three ion-mobility spectra collected for each variety, totaling 12 spectra and establishing an independent test data set. The spectra from this test data set were then tested against a 1D CNN training set comprising 100 ion-mobility spectra (10 replicates per coffee variety). The independent test data set was binned with bin widths of 0.1, 0.15, 0.20, 0.25, and 0.30 ms, and used to test the classification accuracy of the model against the corresponding trained 1D CNN model for each bin width. Surprisingly, we found that data with a 0.10 ms bin width yielded the highest classification accuracy (Figures S20, S21A, and S22). In the CBL coffee bean variety, the probability was below 85% in one replicate, resulting in an accuracy value of 91.66% (Figures S21A and S22). Data with a 0.15 ms bin width yielded 83.33% classification accuracy (Figures S21B and S22), while data with bin widths of 0.20, 0.25, and 0.30 ms yielded 75.00% classification accuracy (Figures S21C-E and S22). As bin width increases, detailed temporal features in the 1D signal become averaged out, reducing class-specific peak resolution and causing overlapping patterns between classes. This loss of information shifts the 1D CNN model from discriminative feature learning (0.10 ms) to under-resolved representations (≥0.20 ms), resulting in systematic misclassification and lower accuracy (Figure S22).

**Figure 5:**
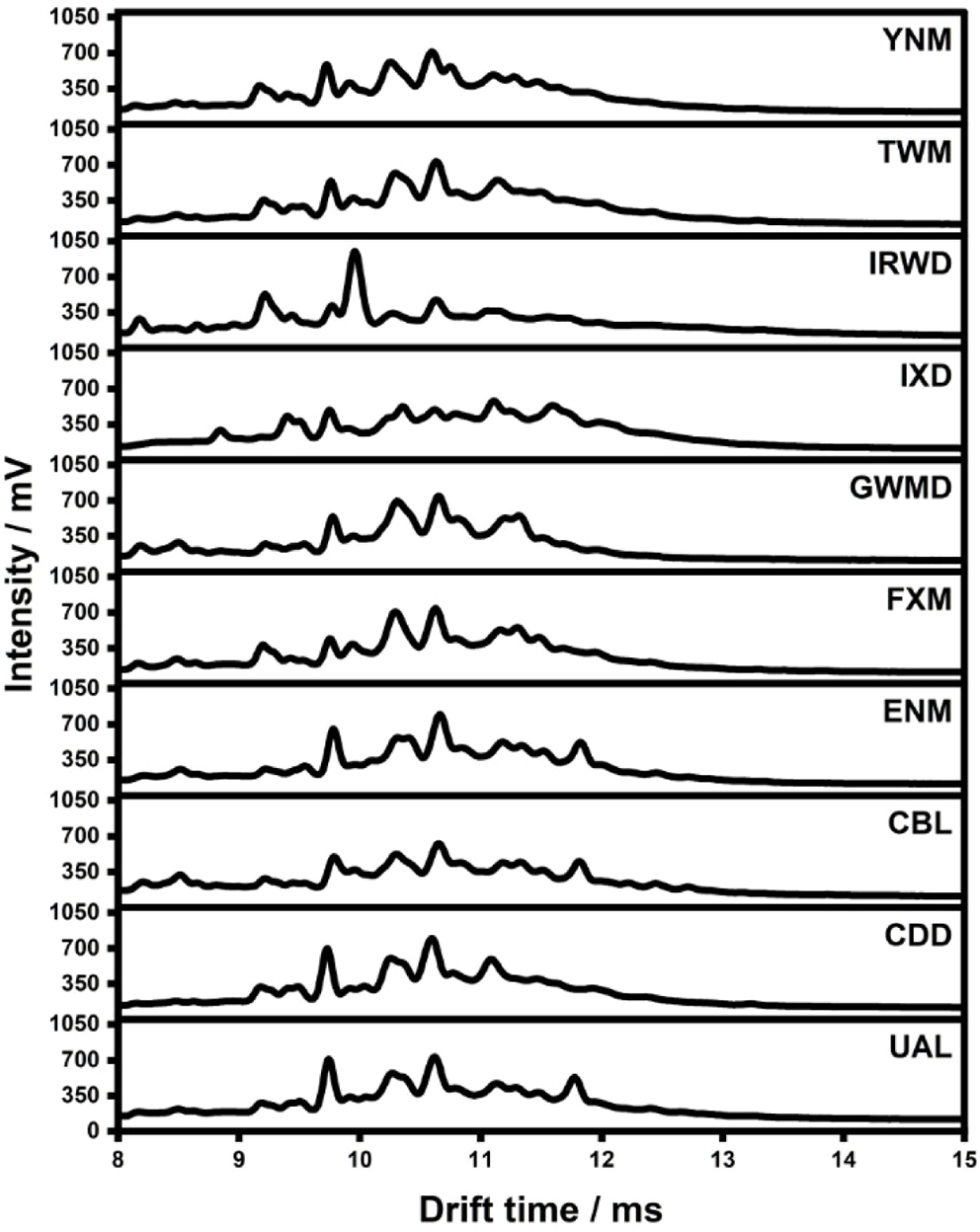
Ion-mobility spectra obtained during hot gas extraction of single coffee beans sampled from 10 coffee varieties.

A limitation of the presented CNN-based classification approach is that the training was performed on replicate measurements of coffee beans originating from the same lot. These measurements capture instrumental noise, small variations in signal alignment, intensity fluctuations, and background changes, thereby introducing variability to which CNN models are expected to be robust. In fact, data augmentation approaches based on slightly perturbed spectra are commonly used in spectroscopic machine-learning workflows to increase the effective training set size when independent samples are limited.^50–52^ However, in future work, to facilitate real-world applications of the method, it would be desirable to perform training using truly independent sample data sets obtained from different coffee lots.

### 3.6 Final Remarks

We have demonstrated a proof-of-concept of a simple analytical approach for chemical fingerprinting of coffee beans, requiring only single-bean samples and employing machine learning for data processing. Custom GUIs were developed to process, visualize, and bin IMS data (Figures S6 and S7). The processed data were successfully differentiated using a custom-built 1D CNN model GUI (Figure S8). The stability of the binned fingerprints (the ninth bin in the data with a 0.25 ms bin width) was verified through statistical analysis, which showed satisfactorily low RSD values ranging from 4.14% to 7.95%. When integrated with a 1D CNN, the platform achieved 100% classification accuracy for four specific varieties at an optimal bin width of 0.25 ms.

The system’s capabilities were further verified by expanding the scope to 10 diverse coffee varieties, in which case the model achieved 91.66% accuracy on an independent test data set with a 0.10 ms bin width. (Samples were excluded if prediction probability was <85%.) Beyond variety classification, the platform proved effective for monitoring aroma degradation over a three-week period and reached 90% accuracy in detecting adulteration. Although the proposed method does not enable identification of specific VOCs, the obtained heuristic readouts still facilitate rapid detection of coffee bean adulteration. In the future, it could also be implemented to classify various solid samples and determine the aging period of meat and cheese products. The online hot-gas extraction setup can also be coupled with MS for the identification of VOCs, but this will undoubtedly increase the capital cost and eliminate portability.

## Supplementary Material



## Notes

Tables S1–S4: model summary for 1D CNN; optimized parameters; probabilities obtained during degradation study; probabilities obtained during adulteration study); Figures S1–S22: photographs of the setup; design and dimensions of the extraction chamber; design and dimensions of heating plates; simplified circuit diagram; flowchart for data processing; screenshots of the custom-designed GUI; optimization spectra; confusion matrix; plot illustrating the model’s validation accuracy; accuracy and loss performance plots; aluminum foil pouches utilized for the degradation study; binned ion-mobility spectra of 10 varieties of coffee beans; confusion matrix for the 10-variety-trained 1D CNN model at different bin width; plot illustrating the model’s test data set accuracy) (PDF)
