# Supplementary material for: Rapid Classification of Coffee Varieties Using Single-Bean Hot Gas Extraction Ion-Mobility Spectrometry with Machine Learning
Source: ACS Meas Sci Au. 2026 Apr 2;6(3):743–51. doi: 10.1021/acsmeasuresciau.6c00039 (PMC13281198; doi:10.1021/acsmeasuresciau.6c00039)
Supplement: Supplementary file 1 [file tg6c00039_si_001.pdf]

SUPPORTING INFORMATION

Rapid Classification of Coffee Varieties Using Single-Bean Hot Gas Extraction Ion-Mobility Spectrometry with Machine Learning

Nathanael Aaron Prayoga,[#] Chamarthi Maheswar Raju,[#] Pawel L. Urban*

Department of Chemistry, National Tsing Hua University

101, Section 2, Kuang-Fu Rd., Hsinchu, 300044, Taiwan

[#] These authors contributed equally to the present work.

* Corresponding author:

P.L. Urban (urban@mx.nthu.edu.tw)

ADDITIONAL TABLES

Table S1. Model summary for 1D CNN.

Layer (type)	Output shape	Parameter #
Conv 1D	(None, 26, 32)	128
Max Pooling 1D	(None, 13, 32)	0
Conv 1D_1	(None, 11, 64)	6208
Max Pooling 1D_1	(None, 5, 64)	0
Flatten	(None, 320)	0
Dense	(None, 128)	41088
Dropout	(None, 320)	0
Dense_1	(None, 64)	8256
Dropout_1	(None, 64)	0
Dense_2	(None, 4)	260

Table S2. Optimized parameters of the single coffee bean hot gas extraction IMS system.

Parameter	Unit	Value
Drift gas	-	nitrogen
Drift gas flow rate	mL min^{-1}	250
Injection pulse width	µs	150
Drift tube temperature	°C	80
Spectrum acquisition time	ms	20
Blocking voltage	V	120
Injection voltage	V	2500
Aperture voltage	V	0
Drift voltage	V	240
Extraction gas flow rate	mL min^{-1}	200
Extraction chamber and gas heater temperature	°C	50
Transfer line	°C	70

Table S3. Probabilities (%) obtained during degradation study of anaerobic fermentation natural Arabica coffee. Predictions were made using a trained 1D CNN model that analyzed four different varieties of coffee beans.

	Test sample No.	Nitrogen flushed sealed	Airtight sealed	Improperly sealed
**Week 0 / fresh**	01	100.00	100.00	100.00
	02	100.00	100.00	100.00
	03	100.00	100.00	100.00
**Week 1**	01	100.00	94.29	0.00
	02	99.99	99.97	0.00
	03	99.96	100.00	0.00
**Week 2**	01	0.00	99.99	0.00
	02	0.00	9.29	0.00
	03	0.00	99.97	0.00
**Week 3**	01	0.00	0.00	0.00
	02	0.00	92.96	0.00
	03	0.00	0.00	0.00

Table S4. Probabilities (%) obtained during adulteration study of Civet Arabica coffee. The 1D CNN model results for Civet Arabica class were used to identify adulterated (unwrapped) and unadulterated (wrapped) coffee beans using the single coffee bean hot gas extraction IMS system. To adulterate Civet Arabica coffee beans, 5 Civet Robusta coffee beans (25%) were mixed with 15 Civet Arabica coffee beans (75%). Note: red denotes a false positive (incorrect identification), whereas green denotes a true positive.

Aluminum foil-unwrapped		Aluminum foil-wrapped	
Test sample No.	Civet Arabica prob. / %	Test sample No.	Civet Arabica prob. / %
01	0.00	01	0.00
02	100.00	02	100.00
03	0.00	03	100.00
04	0.00	04	100.00
05	0.00	05	100.00
06	0.00	06	89.84
07	0.00	07	100.00
08	0.00	08	100.00
09	0.00	09	99.99
10	0.00	10	100.00
11	0.00	11	96.53
12	0.00	12	0.00
13	0.00	13	0.00
14	0.00	14	99.99
15	0.00	15	99.97
16	0.00	16	0.00
17	0.00	17	0.00
18	0.00	18	100.00
19	0.00	19	100.00
20	100.00	20	100.00

ADDITIONAL FIGURES

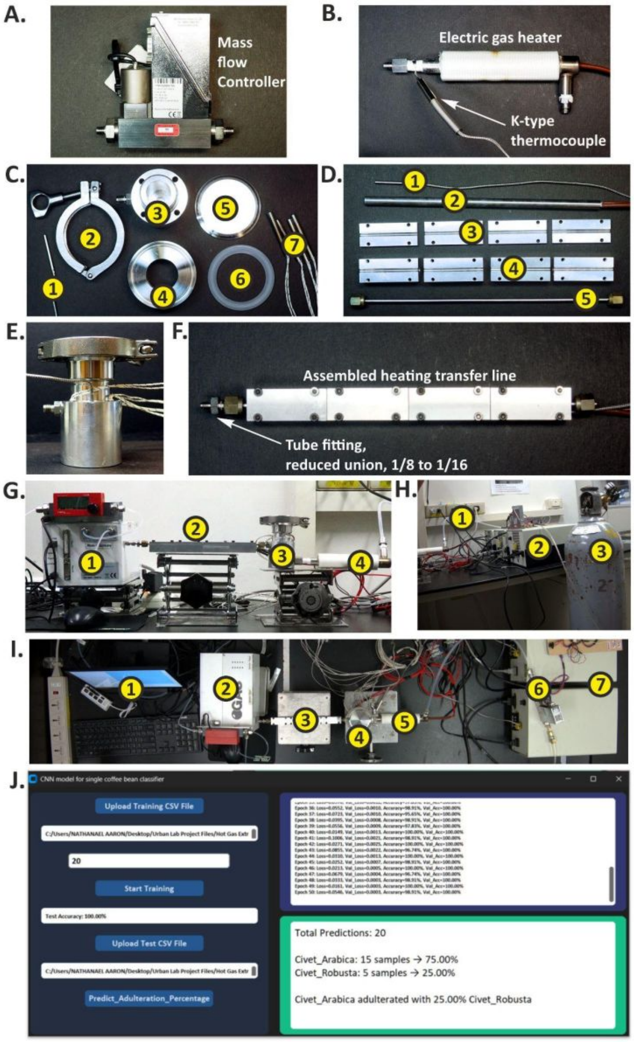

Figure S1. Photographs of the single coffee bean hot gas extraction IMS setup with its elements. (A) mass flow controller; (B) electric gas heater; (C) elements of the sample extraction chamber [(1) k-type thermocouple, (2) 2-inch tri clamp, (3) sample extraction chamber, (4) 1-inch NPT female tri clover, (5) 2-inch end cap, (6) silicone rubber gasket, (7) 4 heating cartridge rods (110 V, 300 W)]; (D) elements of heating transfer line [(1) k-type thermocouple, (2) heating cartridge rod (110 V, 500 W), (3) top heating plates (×4), (4) bottom heating plates, (×4), (5) transfer line]; (E) assembled sample extraction chamber; (F) assembled heating transfer line; (G) close front view of single coffee bean hot gas extraction IMS setup [(1) IMS, (2) heating transfer line, (3) sample extraction chamber, (4) electric gas heater]; (H) [(1) transparent PTFE tube, (2) heating cartridges and electric gas heater power supplies, (3) nitrogen gas cylinder,]; (I) top view of single coffee bean hot gas extraction IMS setup [(1) monitor, (2) IMS, (3) heating transfer line, (4) sample extraction chamber, (5) electric gas heater, (6) mass flow controller, (7) heating cartridges and electric gas heater power supplies]; (J) screenshot of graphical user interface for adulteration test.

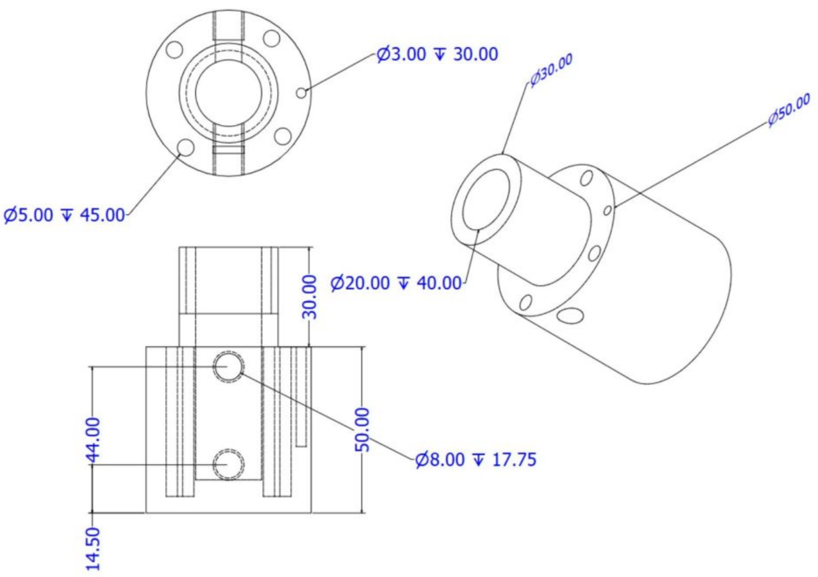

Figure S2. Design and dimensions of the single coffee bean VOCs extraction chamber. Unit: mm.

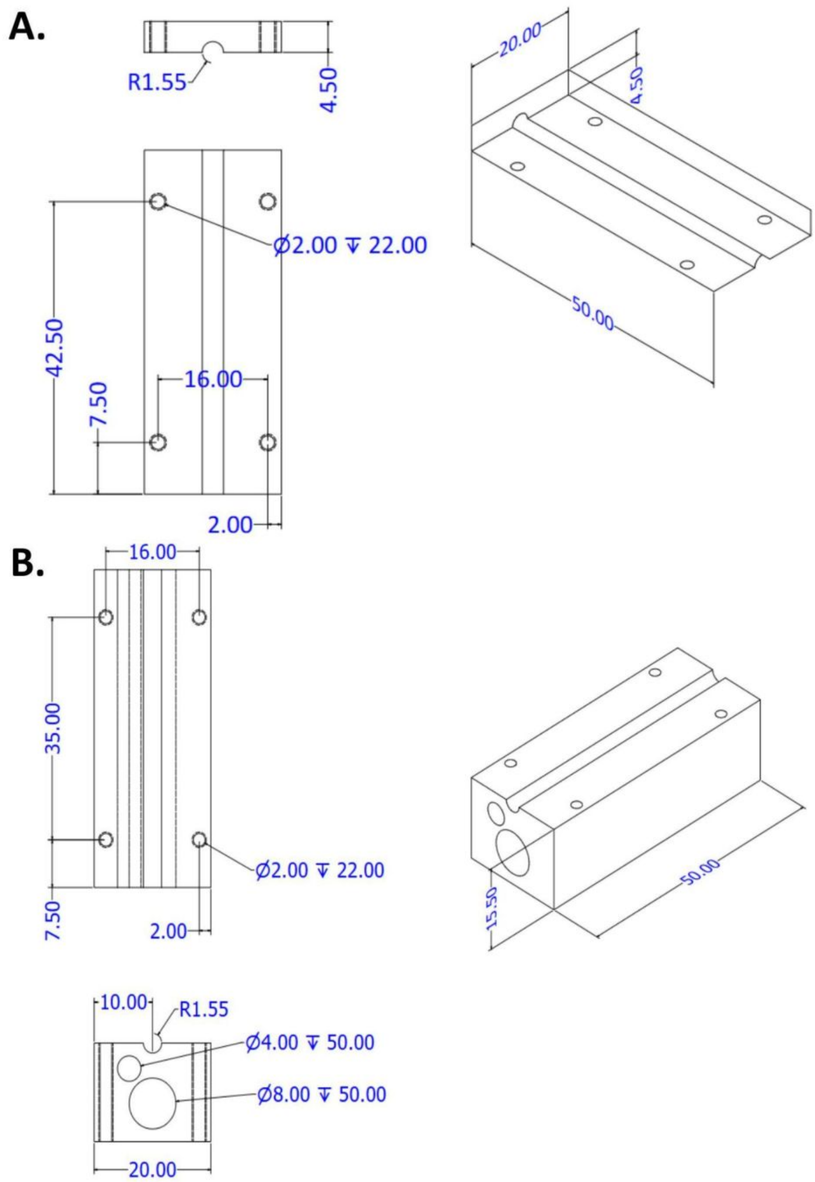

Figure S3. Design and dimensions of single coffee bean VOCs transfer line heating plates: (A) top heating plate; (B) bottom heating plate. Unit: mm.

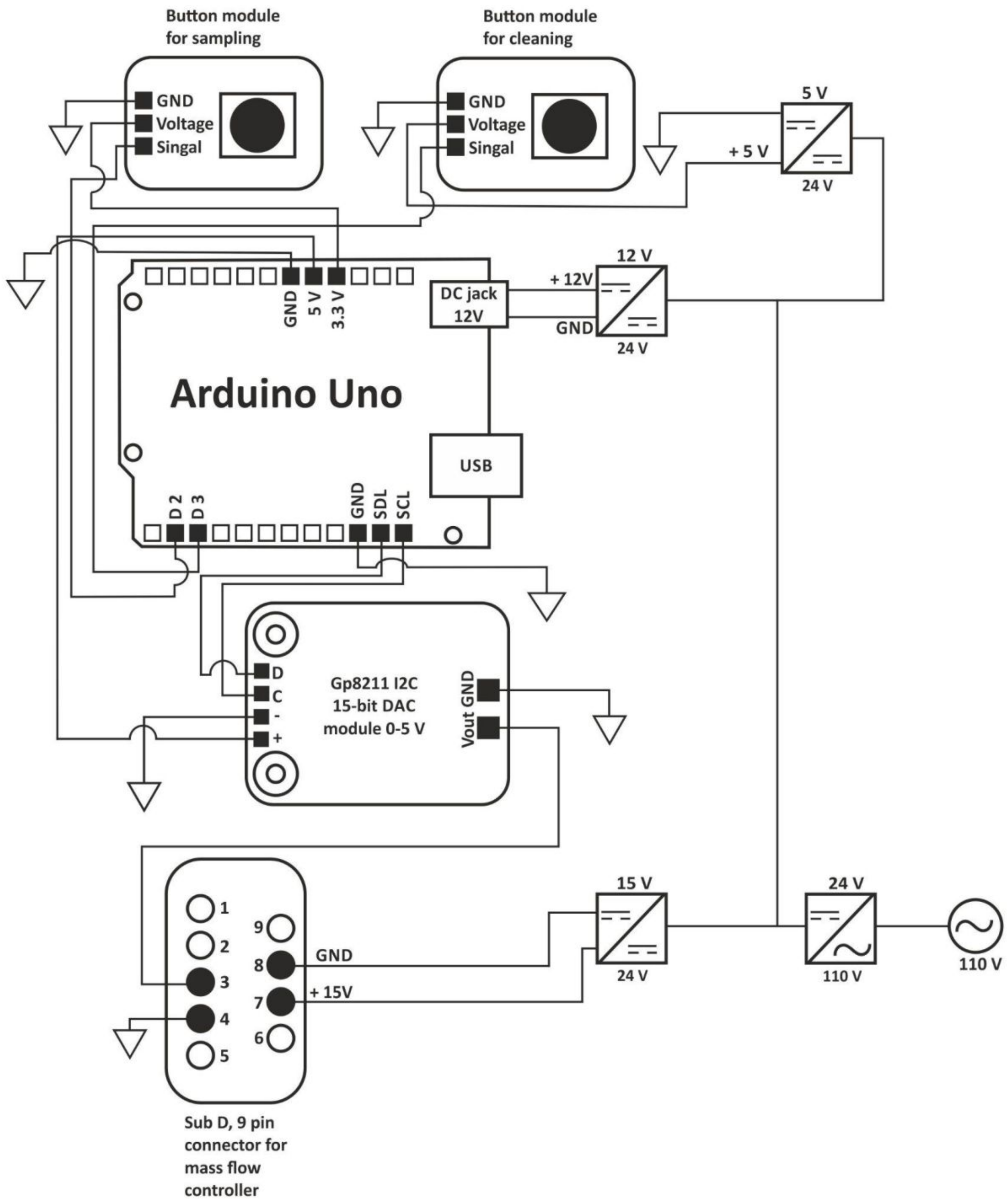

Figure S4. Simplified circuit diagram for a mass flow controller (to control gas flow rate).

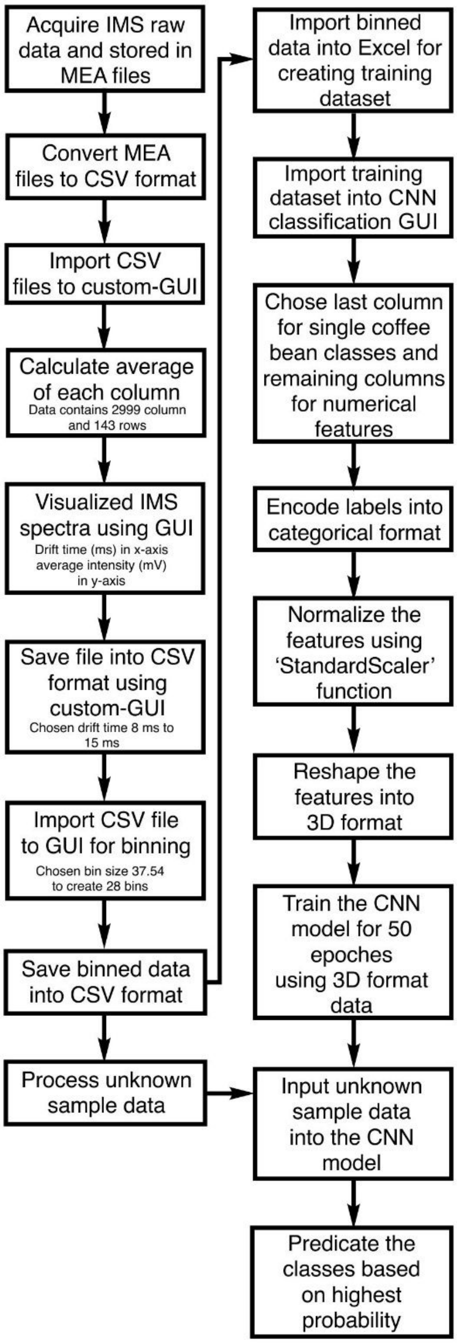

Figure S5. Flow chart for data processing and machine learning.

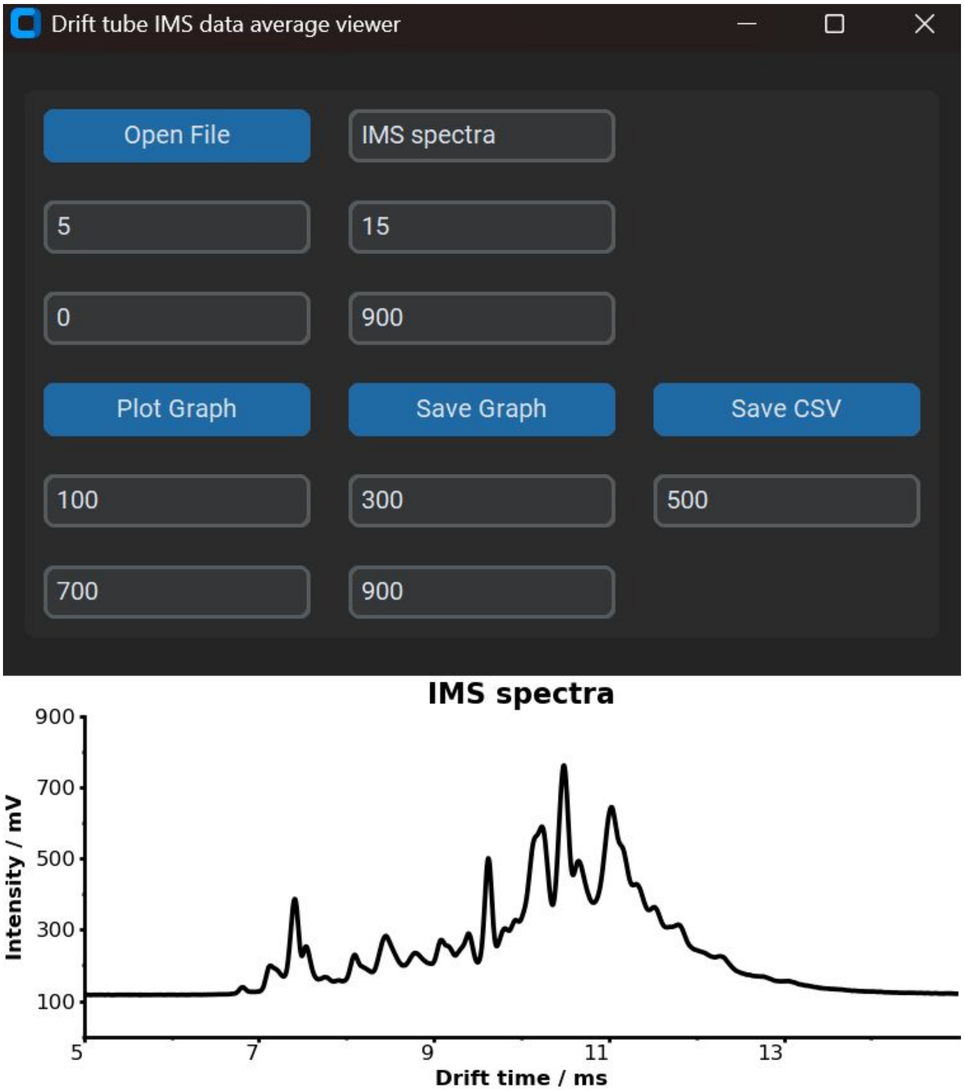

Figure S6. Screenshot of the custom-designed GUI for viewing drift tube IMS data.

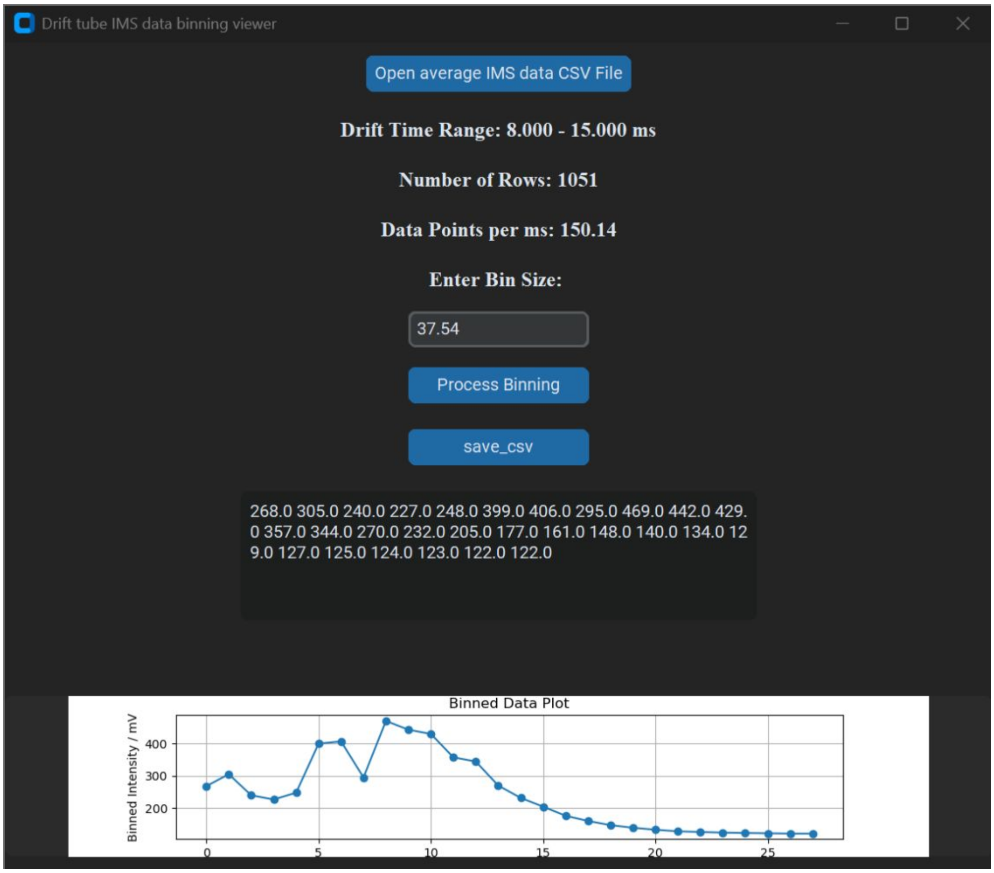

Figure S7. Screenshot of the custom-designed GUI for viewing binned drift tube IMS data.

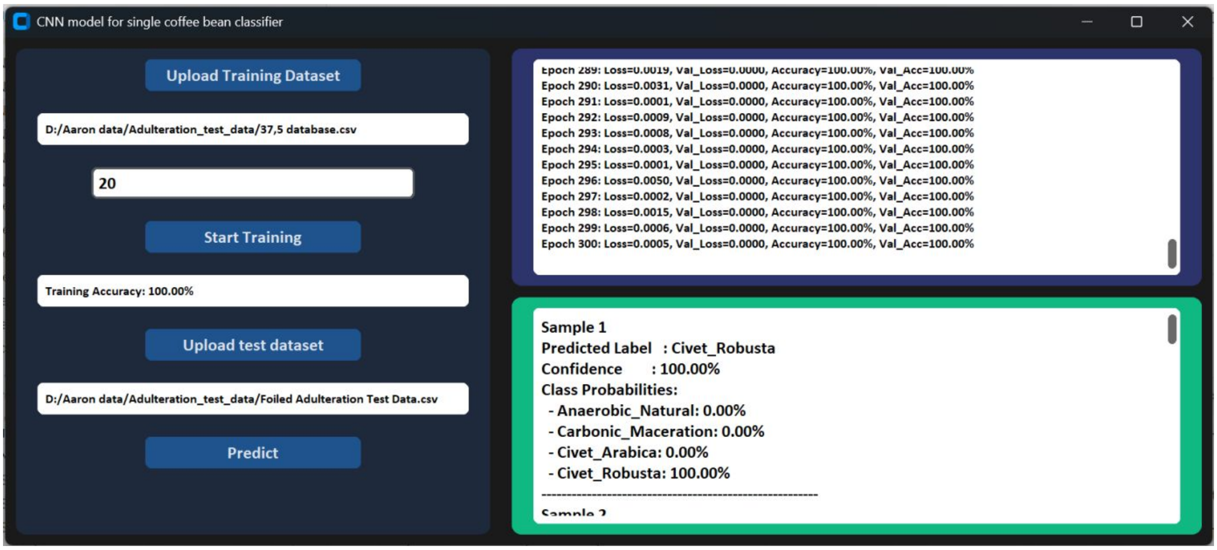

Figure S8. Screenshot of the custom-designed GUI for single coffee bean classification using a 1D CNN model.

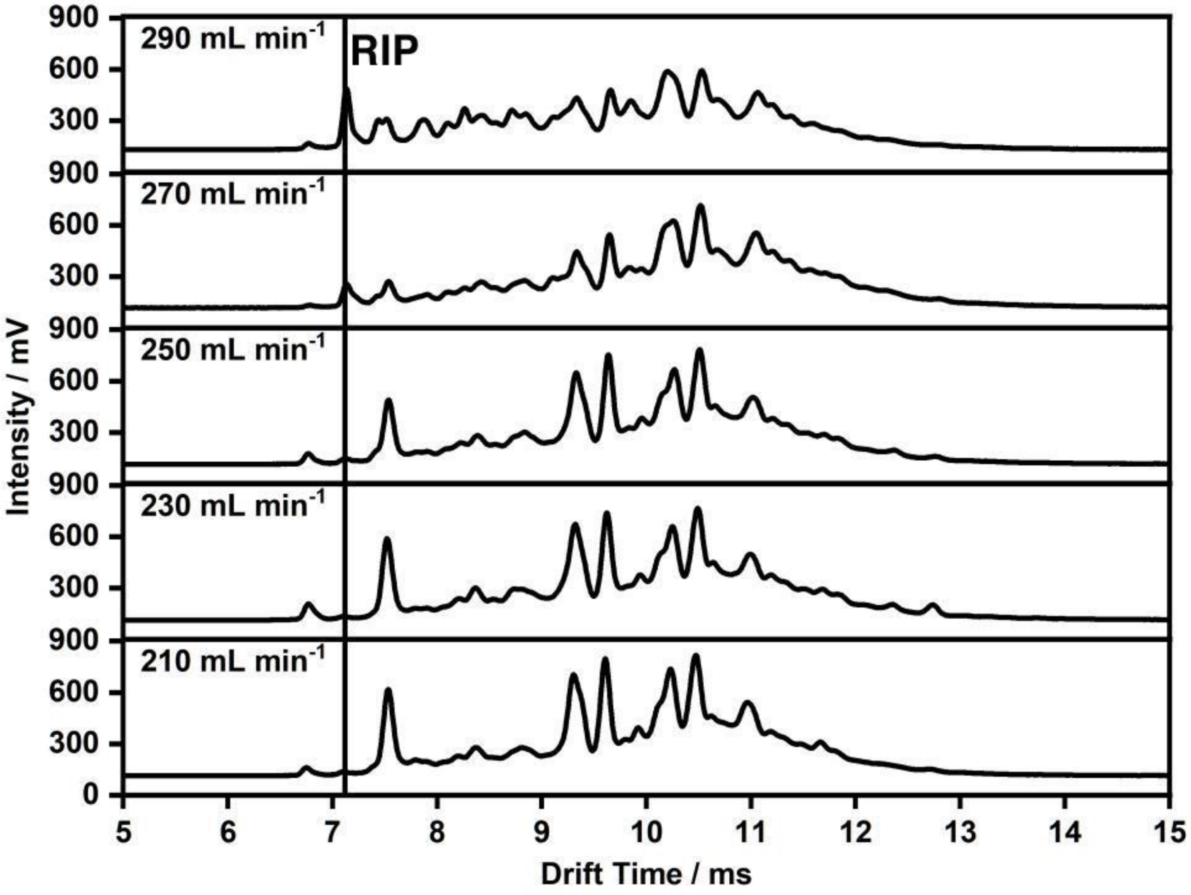

Figure S9. Optimization of the IMS drift gas flow rate. Instrument parameters: spectrum acquisition time, 20 ms; drift voltage, 240 V; blocking voltage, 120 V; injection voltage, 2500 V; injection pulse width, 150 µs; aperture grid voltage, 0 V; drift tube temperature, 80 °C; extraction chamber and gas heater temperature, 50 °C; extraction gas (nitrogen) flow rate, 200 mL min^{-1}; heated transfer line temperature, 70 °C.

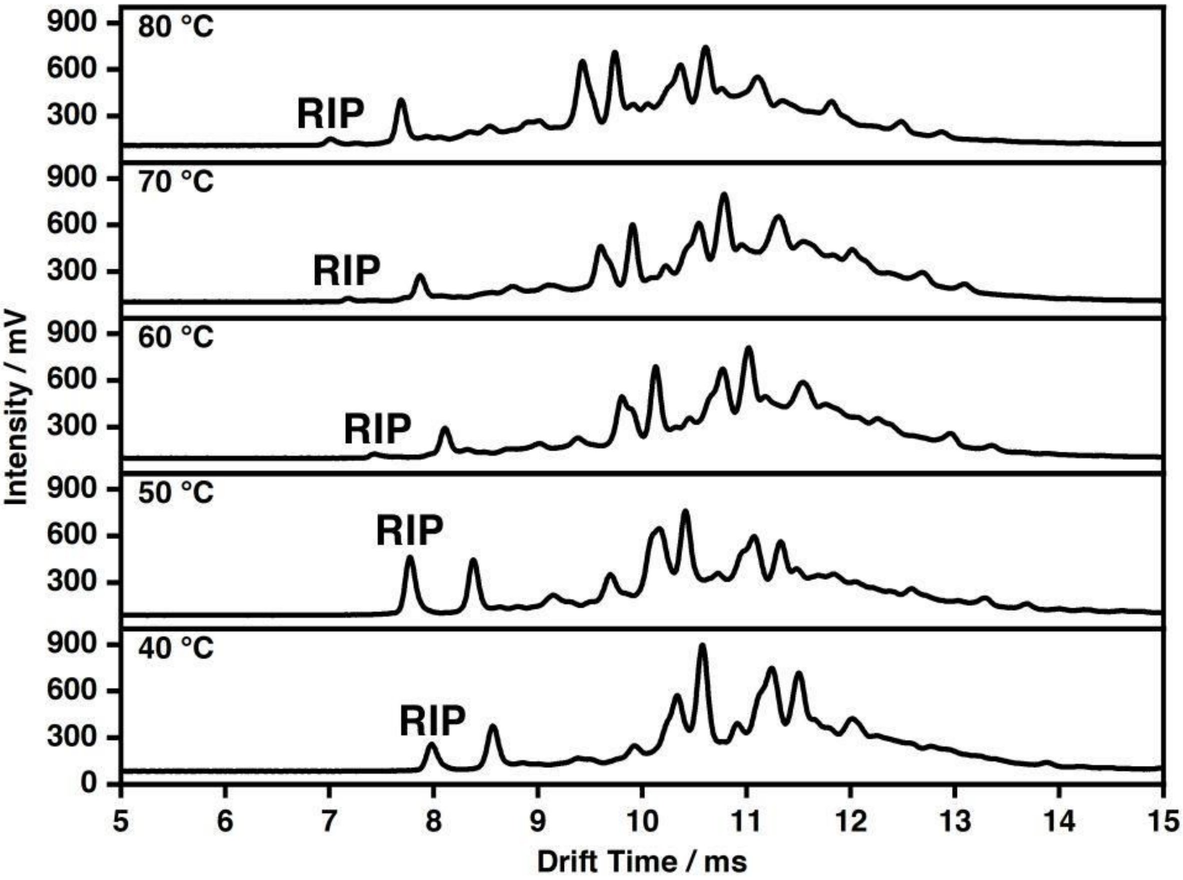

Figure S10. Optimization of the IMS drift tube temperature. Instrument parameters: spectrum acquisition time, 20 ms; drift voltage, 240 V; blocking voltage, 120 V; injection voltage, 2500 V; injection pulse width, 150 µs; aperture grid voltage, 0 V; drift gas flow rate, 250 mL min^{-1}; extraction chamber and gas heater temperature, 50 °C; extraction gas (nitrogen) flow rate, 200 mL min^{-1}; heated transfer line temperature, 70 °C.

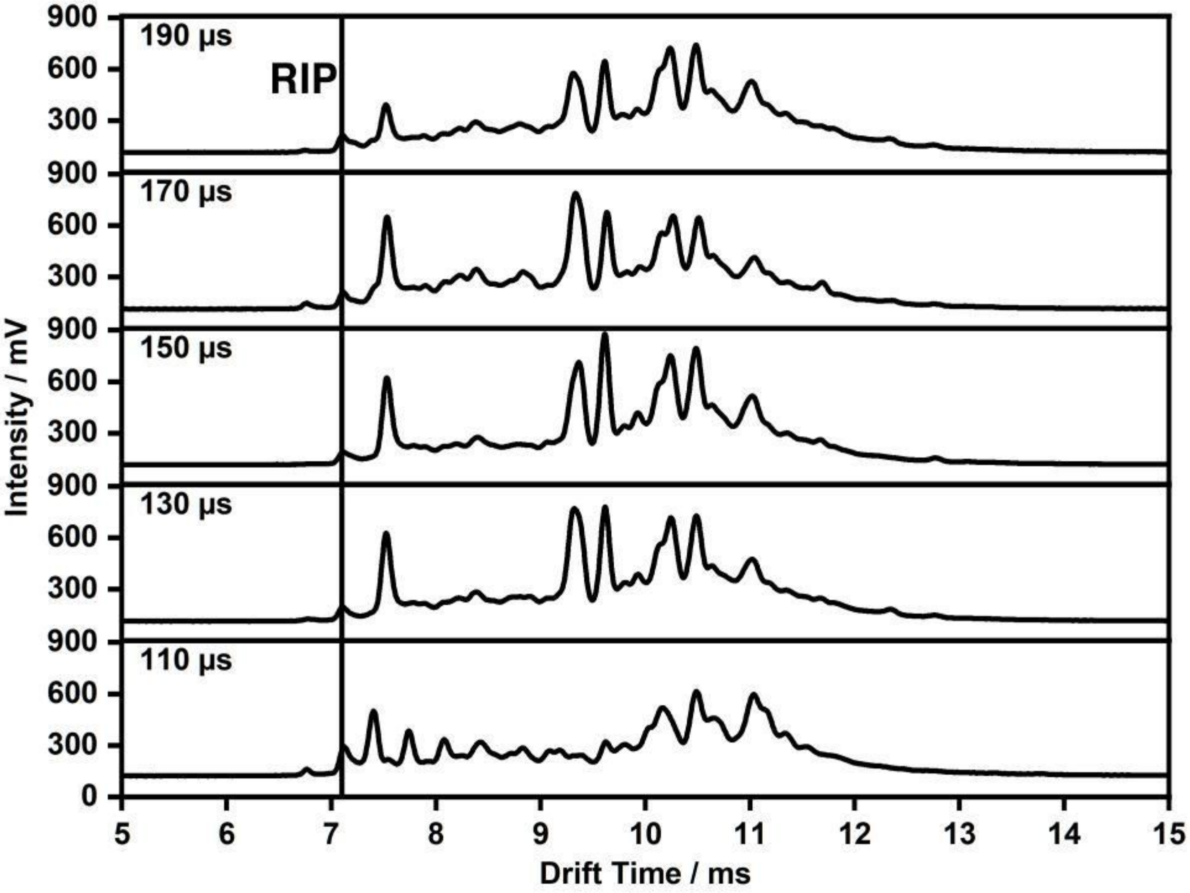

Figure S11. Optimization of the IMS injection pulse width. Instrument parameters: spectrum acquisition time, 20 ms; drift voltage, 240 V; blocking voltage, 120 V; injection voltage, 2500 V; aperture grid voltage, 0 V; drift gas flow rate, 250 mL min^{-1}; drift tube temperature, 80 °C; extraction chamber and gas heater temperature, 50 °C; extraction gas (nitrogen) flow rate, 200 mL min^{-1}; heated transfer line temperature, 70 °C.

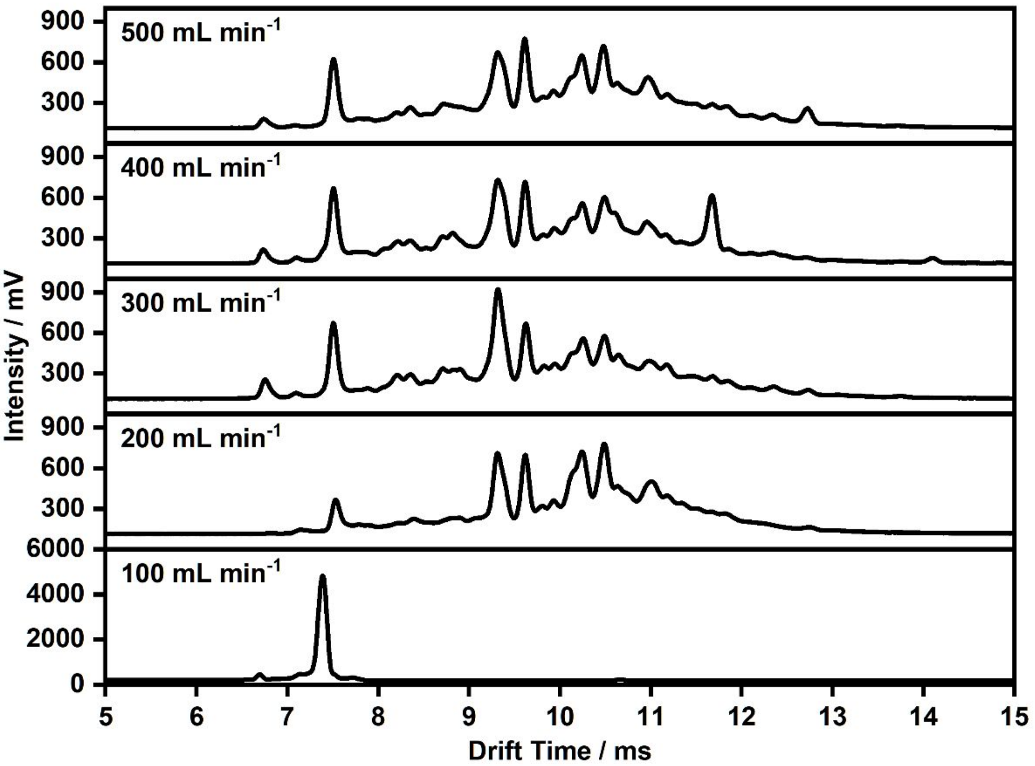

Figure S12. Optimization of the extraction gas (nitrogen) flow rate. Instrument parameters: spectrum acquisition time, 20 ms; drift voltage, 240 V; blocking voltage, 120 V; injection voltage, 2500 V; aperture grid voltage, 0 V; injection pulse width, 150 µs; drift gas flow rate, 250 mL min^{-1}; drift tube temperature, 80 °C; extraction chamber and gas heater temperature, 50 °C; heated transfer line temperature, 70 °C.

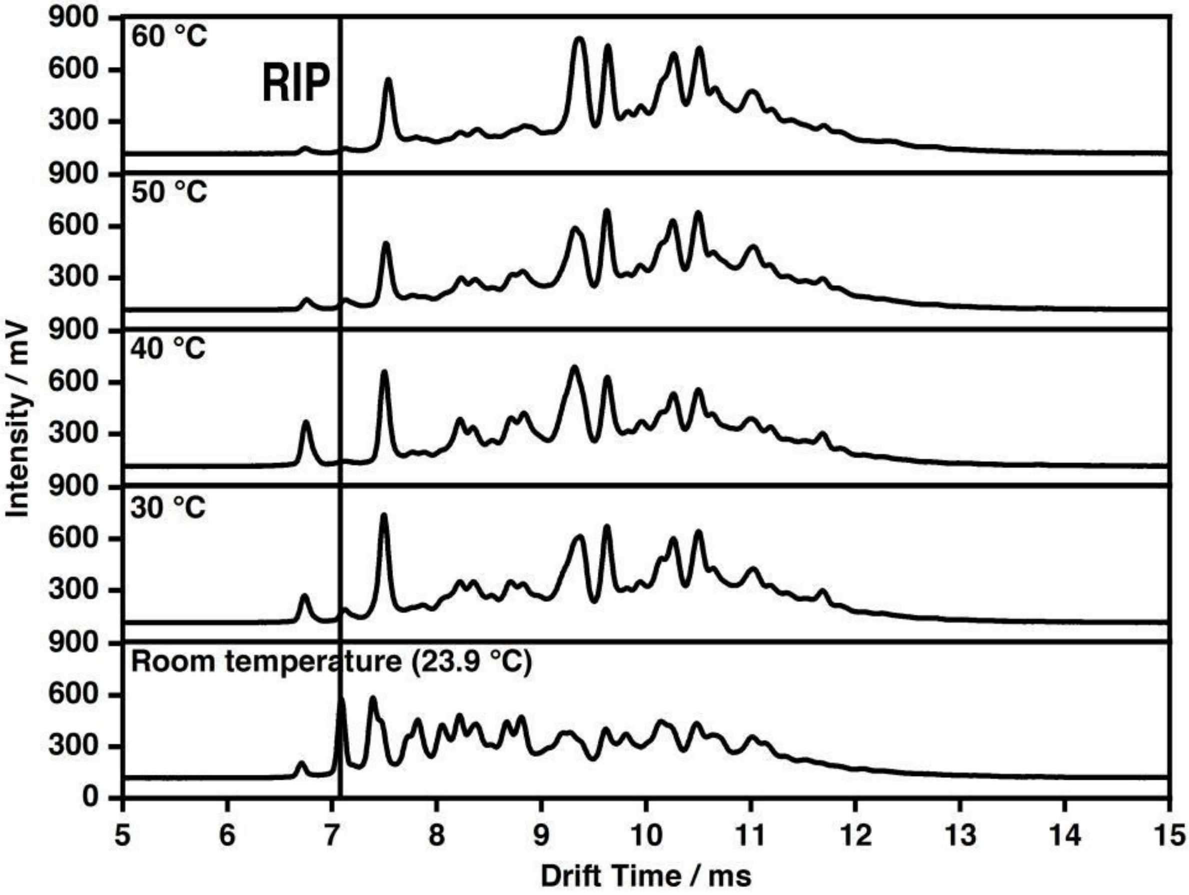

Figure S13. Optimization of the extraction chamber and heater temperature. Instrument parameters: spectrum acquisition time, 20 ms; drift voltage, 240 V; blocking voltage, 120 V; injection voltage, 2500 V; aperture grid voltage, 0 V; injection pulse width, 150 µs; drift gas flow rate, 250 mL min^{-1}; drift tube temperature, 80 °C; extraction gas (nitrogen) flow rate, 200 mL min^{-1}; heated transfer line temperature, 70 °C.

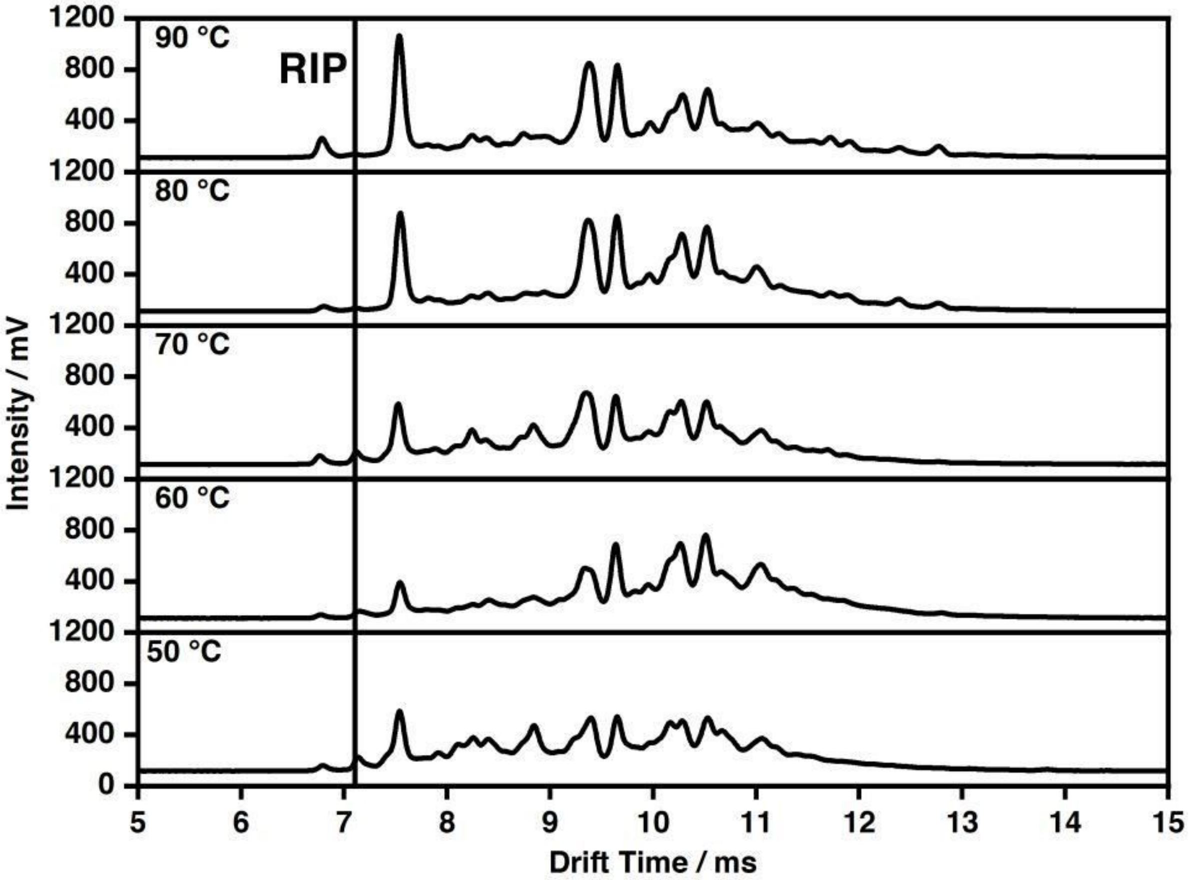

Figure S14. Optimization of the heated transfer line temperature. Instrument parameters: spectrum acquisition time, 20 ms; drift voltage, 240 V; blocking voltage, 120 V; injection voltage, 2500 V; aperture grid voltage, 0 V; injection pulse width, 150 µs; drift gas flow rate, 250 mL min^{-1}; drift tube temperature, 80 °C; extraction chamber and gas heater temperature, 50 °C; extraction gas (nitrogen) flow rate, 200 mL min^{-1}.

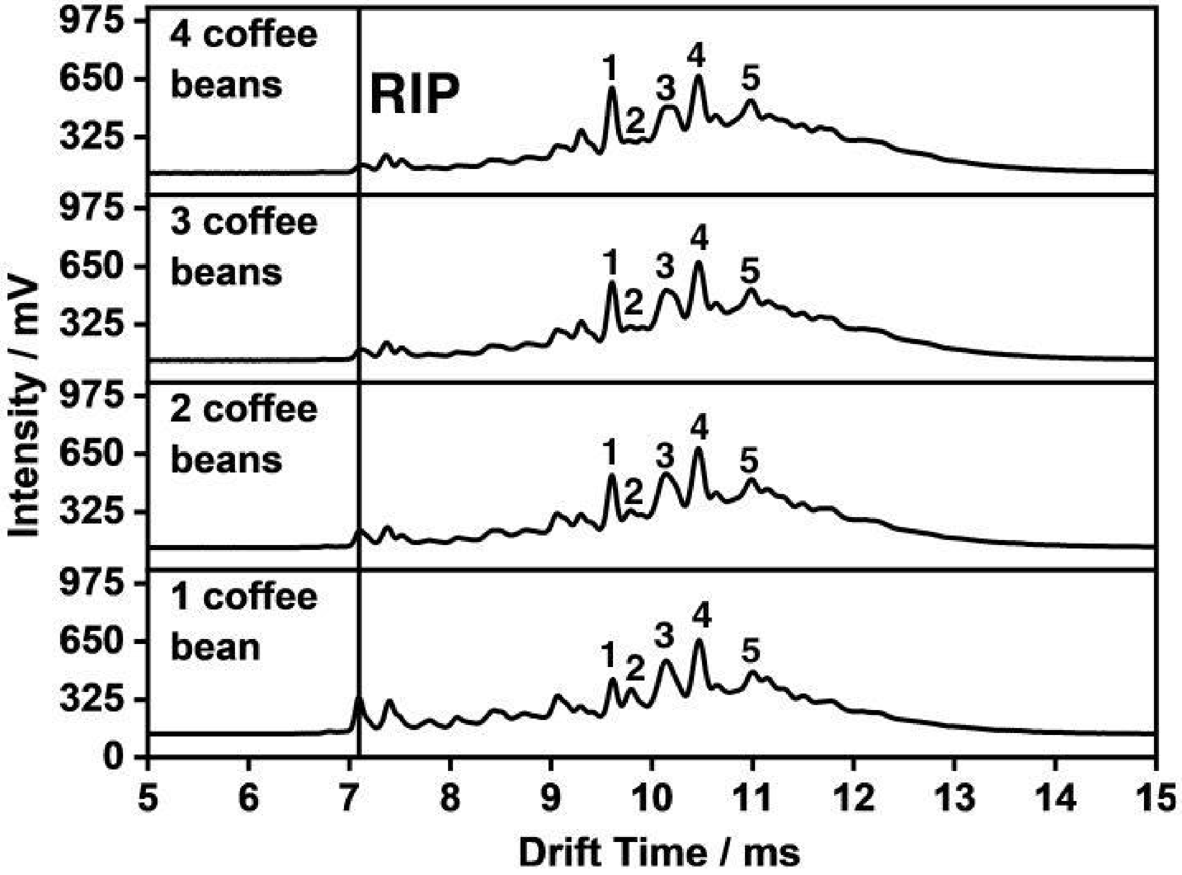

Figure S15. Optimization of the appropriate quantity of coffee beans. For instrumentation parameters, refer to **Table S2**.

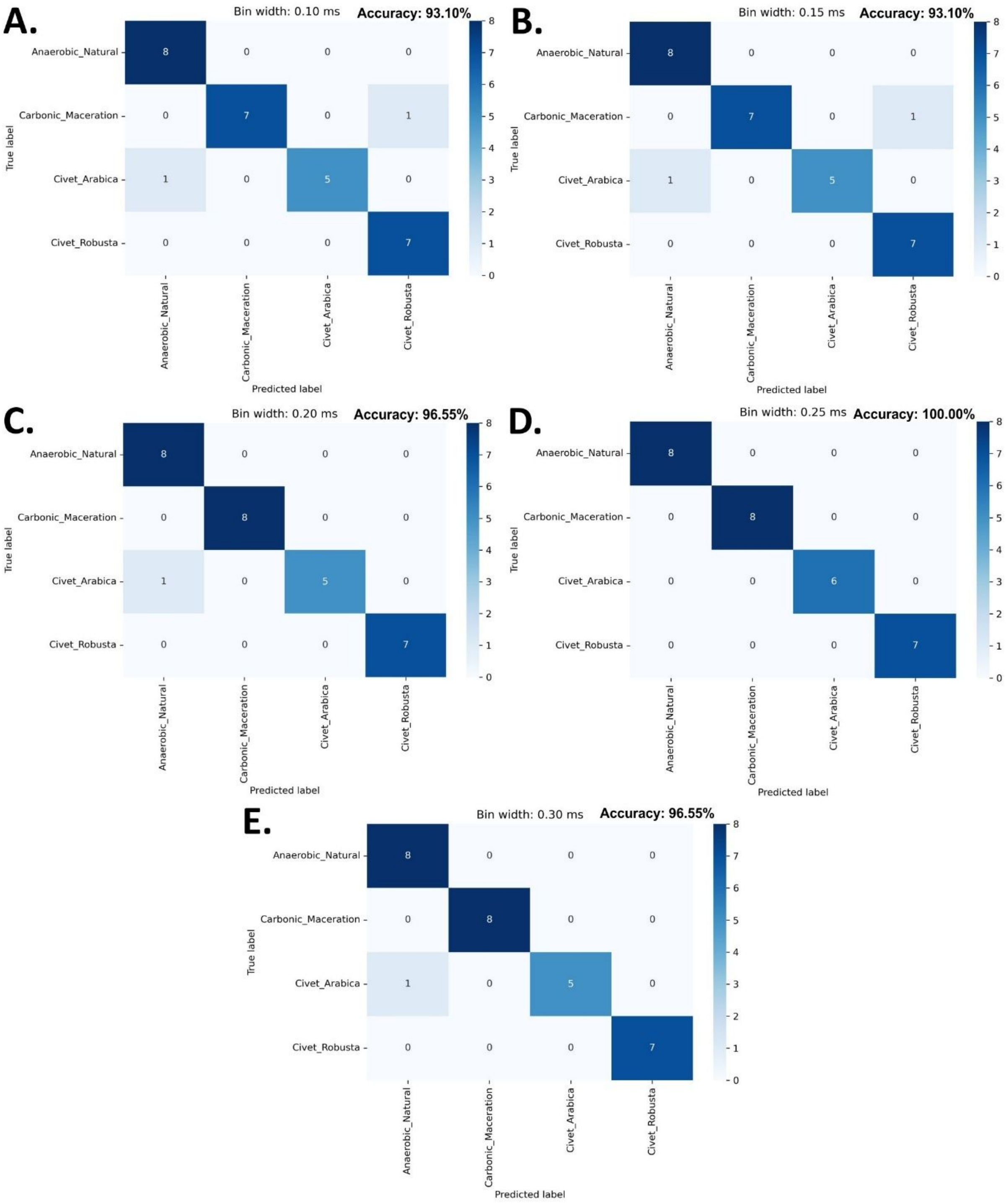

Figure S16. Confusion matrices for the 1D CNN model used to train and classify/validate four coffee bean varieties (anaerobic fermentation natural Arabica, carbonic maceration Arabica, Civet Arabica, and Civet Robusta) at different bin widths.

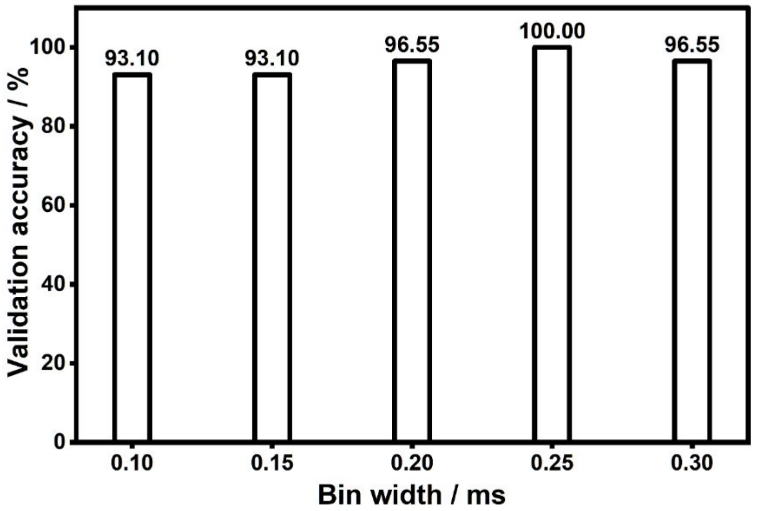

Figure S17. A plot illustrating the model's validation accuracy derived from a confusion matrix for a 1D CNN across various bin widths, involving four coffee varieties.

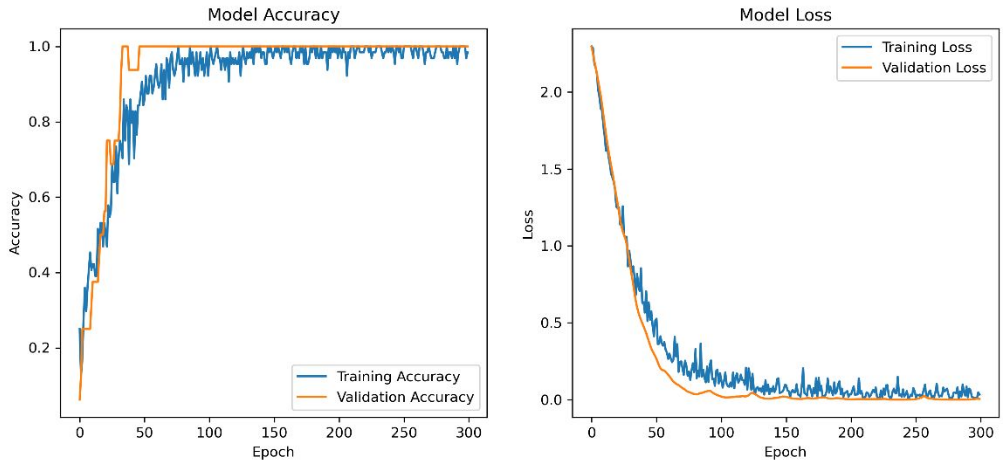

Figure S18. Accuracy and loss performance graphs for the 1D CNN model over 300 epochs with a bin width of 0.25 ms.

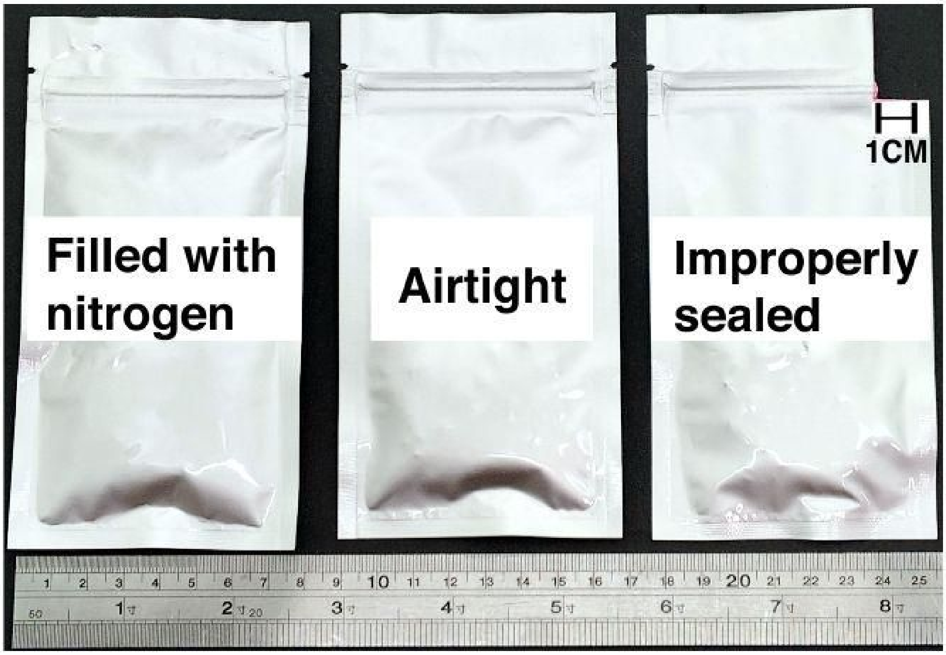

Figure S19. Photograph of aluminum foil pouches utilized for the degradation study.

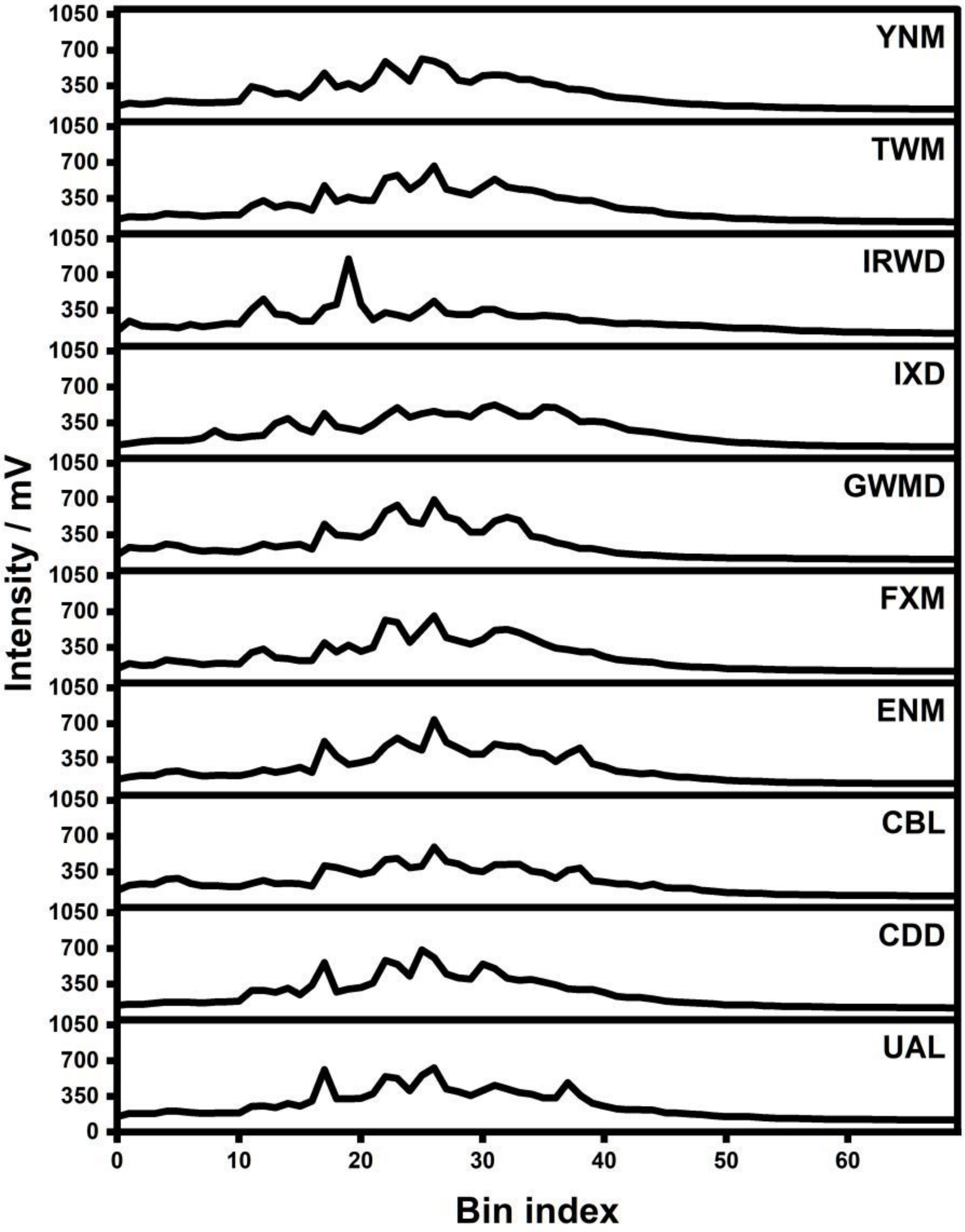

Figure S20. Binned (0.1 ms bin width) ion mobility spectra obtained during hot gas extraction IMS analysis of single coffee beans pertaining to 10 varieties.

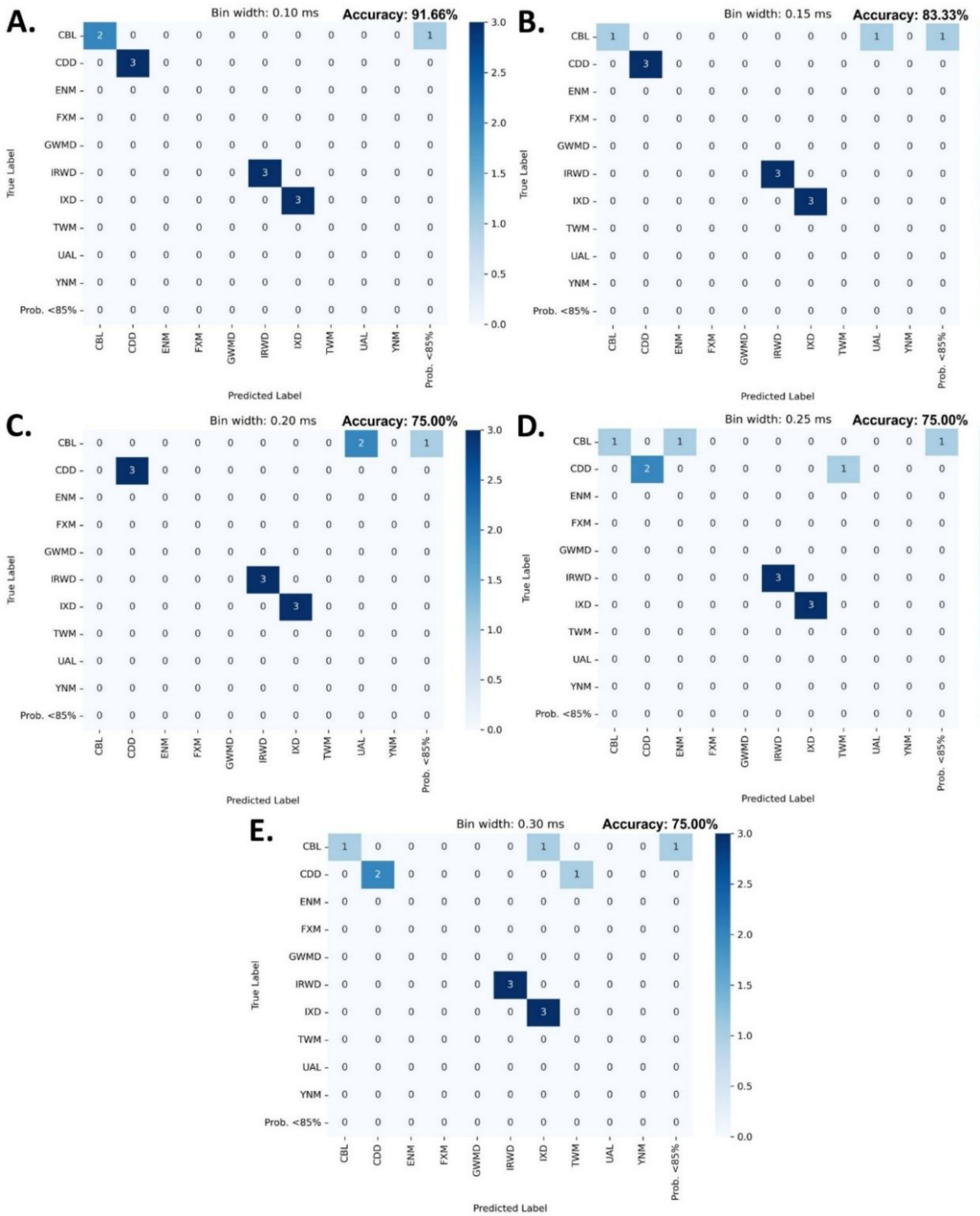

Figure S21. Confusion matrices for classifying four selected coffee bean varieties, characterized with different processes and origins (CBL, CDD, IRWD, and IXD), tested against the 10-variety-trained 1D CNN model at various bin widths.

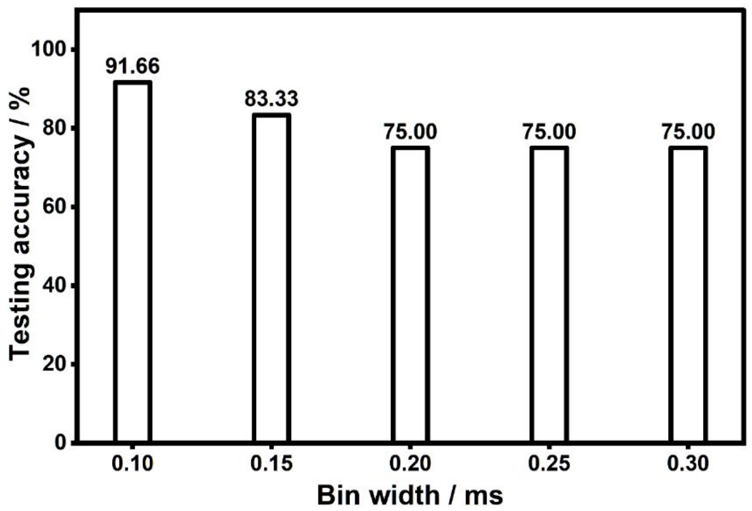

Figure S22. A plot illustrating the model's test dataset accuracy derived from a confusion matrix (**Figure S21**) for a 1D CNN across various bin widths, involving four selected coffee bean varieties characterized with different processes and origins (CBL, CDD, IRWD, and IXD).
